# Comprehensive Analysis of Antimicrobial, Heavy Metal, and Pesticide Residues in Commercial Organic Fertilizers and Their Correlation with Tigecycline-Resistant *tet*(X)-Variant Genes

**DOI:** 10.1128/spectrum.04251-22

**Published:** 2023-03-14

**Authors:** Tao He, Jun Li, Lan Gong, Yang Wang, Ruichao Li, Xing Ji, Fengting Luan, Minmin Tang, Lei Zhu, Ruicheng Wei, Ran Wang

**Affiliations:** a Jiangsu Key Laboratory for Food Quality and Safety—State Key Laboratory Cultivation Base of Ministry of Science and Technology, Institute of Food Safety and Nutrition, Jiangsu Academy of Agricultural Sciences, Nanjing, China; b Key Laboratory of Animal Antimicrobial Resistance Surveillance, Ministry of Agriculture and Rural Affairs, College of Veterinary Medicine, China Agricultural University, Beijing, China; c College of Veterinary Medicine, Yangzhou University, Yangzhou, China; Yangzhou University

**Keywords:** antimicrobial resistance, chemical contaminants, organic fertilizer, plasmid conjugation, type IV secretion system

## Abstract

With the issue of the antimicrobial additive ban in feed in Chinese animal husbandry, it is important to determine the potential drivers of the spread of the newly discovered tigecycline-resistant *tet*(X)-variant genes. Here, we investigated the correlations between residues of heavy metals, antimicrobials, and pesticides and the relative abundance of *tet*(X)-variant genes in 94 commercial organic-fertilizer samples collected from 9 Chinese provinces. A total of 5 heavy metals (mercury, lead, arsenic, chromium, and cadmium), 10 antimicrobials, and 18 pesticides were detected. The *tet*(X)-variant genes, including *tet*(X)/(X2), *tet*(X3), *tet*(X4), *tet*(X5), and *tet*(X6) were detected in 39 (41.5%) samples. Although *tet*(X)-variant-carrying bacteria were not isolated from these samples, the *tet*(X4)-carrying plasmids could be captured by exogenous Escherichia coli. Correlation analysis revealed that heavy metals, other than antimicrobials, showed a significant positive association with the relative abundance of the *tet*(X)-variant genes, especially *tet*(X3) and *tet*(X4) (*R* = 0.346 to 0.389, *P < *0.001). The correlation was attributed to the coselection of the *tet*(X3)/*tet*(X4) gene on the same plasmid and the conjugation-promoting effect of *tet*(X3)/*tet*(X4)-carrying plasmids by subinhibitory concentrations of heavy metals. The heavy metals increased the permeability of the bacterial outer membrane and upregulated the transcription of type IV secretion system (T4SS)-encoding genes on *tet*(X)-variant-carrying plasmids, therefore enhancing the bacterial conjugation rates. Taken together, our findings have indicated that heavy metals may play an important role in spreading *tet*(X)-variant genes within the animal manure-related environment.

**IMPORTANCE** An antimicrobial resistance gene (ARG) is considered a novel contaminant for the environment. Most animal feces are usually made into commercial organic fertilizers in China and will pose a threat to the farmland soil and agricultural product if fertilizers harboring clinically significant antimicrobial-resistant (AMR) genes are applied on farmland. This study has indicated that heavy metals may play an important role in the transmission of transferable tigecycline resistance genes [*tet*(X3) and *tet*(X4)]. The mechanism was that heavy metals posed a coselection effect of the *tet*(X3)/*tet*(X4) gene on the same plasmid and could increase the conjugation ability of *tet*(X3)/*tet*(X4)-carrying plasmids. The conjugation-promoting concentrations of heavy metals are lower than the maximal limits defined in the national standard for fertilizers, indicating a high transmission risk of *tet*(X3)/*tet*(X4) genes within the animal manure-related environment. The findings in this study will provide scientific evidence for the future development of effective measures to reduce AMR dissemination.

## INTRODUCTION

Tigecycline is one of the most promising last-resort antimicrobials because it can treat serious bacterial infections, especially those caused by extensively drug-resistant (XDR) *Enterobacteriaceae* and Acinetobacter in humans (1, 2). The most common mechanisms of tigecycline resistance are conferred by the overexpression of nonspecific active efflux pumps or mutations within the drug-binding sites in the ribosome (3). Since the discovery of *tet*(X3) and *tet*(X4) in *Enterobacteriaceae* and Acinetobacter spp. from animals, animal-derived food, and humans, it has caused great public concern, as *tet*(X)-variant genes can facilitate transmissible high-level tigecycline resistance (4, 5). *Tet*(X) is the first reported tetracycline-enzymatic-modification gene encoding the monooxygenase enzyme (6). To date, five functional variants of *tet*(X) have been identified: *tet*(X2), *tet*(X3), *tet*(X4), *tet*(X5), and *tet*(X6) (7–9). An expanded pool of *tet*(X)-like genes [*tet*(X7) to *tet*(X16)] has been identified and characterized in environmental and human commensal metagenomes by the antimicrobial selection of metagenomic libraries (10). Of these variants, *tet*(X) and *tet*(X2) are found in *Bacteroides* spp. (obligate anaerobes) but are nontransferable (9), whereas the plasmid-borne *tet*(X3), *tet*(X4), *tet*(X5), and *tet*(X6) are widely identified in bacteria from animals, posing a threat to animal-derived food safety and global public health (4, 7, 8).

China is one of the largest domestic animal producers in the world, and animal feces constitute a large reservoir of antimicrobial resistance genes (ARGs) (11, 12). The application of animal manures on farmlands represents a major pathway for introducing ARGs into agricultural soils and manure-fertilized products (e.g., vegetables) (13, 14). Our previous study revealed that the *tet*(X)-variant genes, including *tet*(X)/(X2), *tet*(X3), and *tet*(X4), could be transmitted from chicken manure to soil and lettuce and highlighted the contribution of veterinary antimicrobials, such as tetracyclines, on the spread of the *tet*(X)-variant genes (15). To constrain the development of animal-derived bacterial resistance, the Chinese Ministry of Agriculture and Rural Affairs issued Announcement No. 194 in 2019 to withdraw all growth-promoting antimicrobial additives in feed except Chinese herbal medicine as of 1 January 2020 (http://www.moa.gov.cn/nybgb/2019/201907/202001/t20200103_6334292.htm). As the drug feed additives accounted for ~50% of all veterinary antimicrobial consumption in the Chinese market before 2020 (16), the impact of veterinary antimicrobials on the distribution of ARGs along the food chain would be largely decreased.

To date, investigations have been focused on the prevalence of ARGs in animal manures and their correlation with the antimicrobial residues, which are considered the main driver for the spread of ARGs (13, 15, 17, 18). In addition to antimicrobials, other potential drivers, such as heavy metals, which inevitably contaminated animal feed and were persistently present in animal feces, should not be neglected under the drug-additive ban in China (19, 20). Pesticides are also common chemical contaminants in organic fertilizers, as some pesticides are widely used in farmland to control plant diseases and pests (21). In recent years, a nationwide campaign against pollutants from the intensive livestock industry has driven the production and sale of commercial organic fertilizers from animal farms. Compared with manure, commercial organic fertilizer is harmless and plays a significant role in the sustainable development of agriculture (22). With rising consumer demand for organic food in China, the use of commercial organic fertilizers is increasing. However, the chemical contaminants, including heavy metals, antimicrobials, and pesticides, in commercial fertilizers and the potential selective effect of these chemical contaminants for disseminating clinically important resistance genes, such as *tet*(X)-variants, were not clear in China. Thus, this study aimed to investigate the residual levels of three chemical contaminants (heavy metals, antimicrobials, and pesticides) in commercial organic fertilizers collected from nine provinces in China. In addition, the occurrence and relative abundance of various *tet*(X)-variant genes [*tet*(X) and *tet*(X2) to *tet*(X6)] were investigated, and their associations with the detected levels of heavy metals, antimicrobials and pesticide residues were analyzed. The potential mechanisms of heavy metals to promote the transmission of *tet*(X)-variant genes were further elucidated.

## RESULTS

### Concentrations of heavy metal, antimicrobial, and pesticide residues.

**FIG 1:**
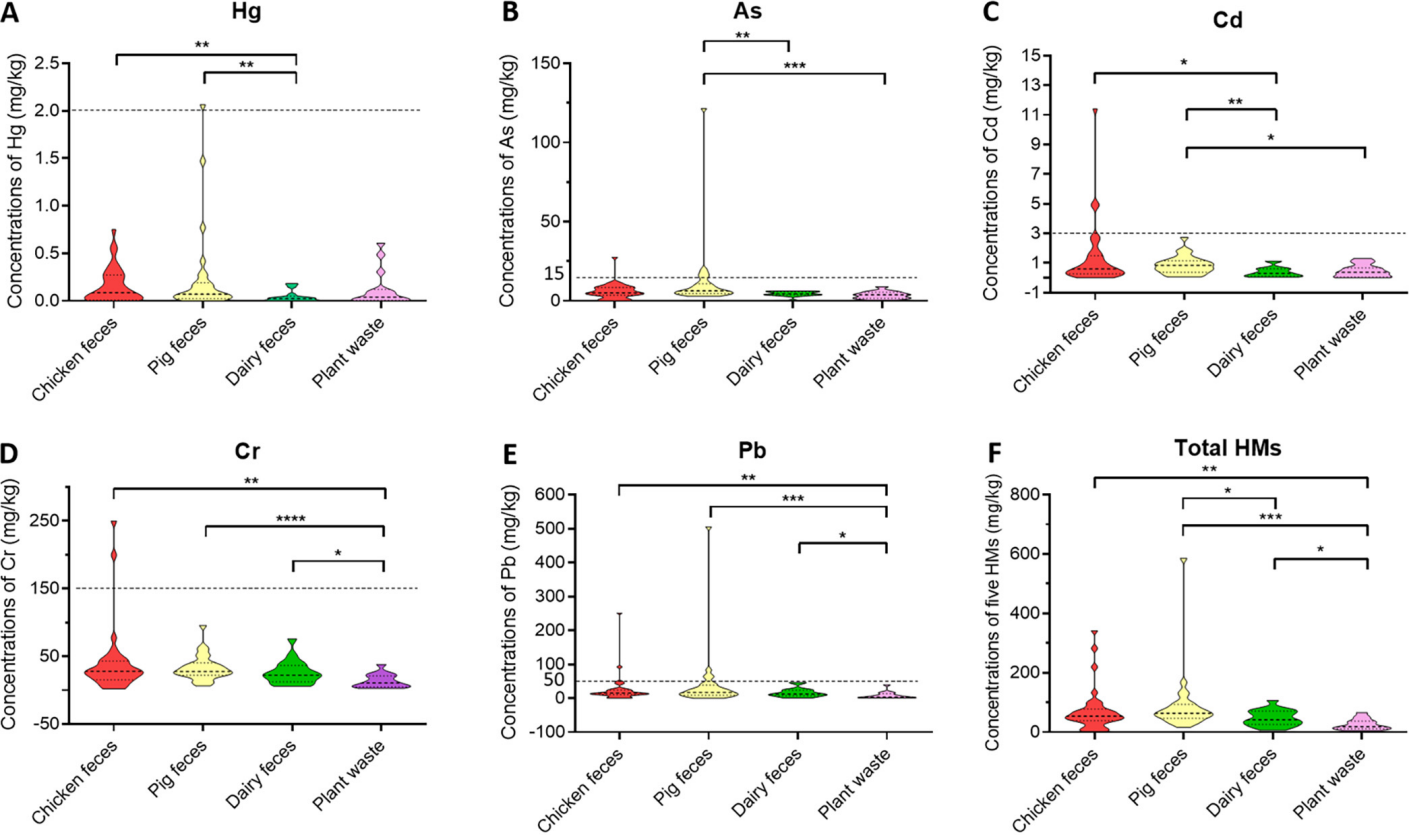
Comparison of contents of heavy metals in organic fertilizer samples of different origins shown on a violin plot. (A to F) Comparison of contents of Hg, As, Cd, Cr, and Pb and totals of five heavy metals (HMs) in samples, respectively. The *x* axis represents the fertilizer samples of different origins. The dotted lines indicate the maximum limits of the heavy metals set in the Chinese national standard NY 525-2012. An unpaired *t* test was used to perform statistical significance; *, *P < *0.05, two-tailed; **, *P < *0.01, two-tailed; ***, *P < *0.001, two-tailed; ****, *P < *0.0001, two-tailed.

As shown in Table S5 in the supplemental material, all 94 samples contained As, Cd, Cr, and Pb, and 73 (77.7%) samples harbored Hg. The detectable concentrations of heavy metals were identified, including As (0.03 to 39.56 mg/kg), Cd (0.01 to 11.42 mg/kg), Cr (2.19 to 250 mg/kg), Pb (0.23 to 251.19 mg/kg), and Hg (0 to 2.07 mg/kg) (Table 1 and Table S5). There were 17 (18.09%) samples in which the content of at least one heavy metal exceeded the corresponding maximum limit in the Chinese national standard NY 525-2012. Of the five heavy metals, Pb was detected with the highest ratio exceeding the standard (6.38%, 6/94), followed by As (5.32%, 5/94), Cd (3.19%, 3/94), Cr (2.13%, 2/94), and Hg (1.06%, 1/94). The highest concentrations of Pb, Cd, As, Cr, and Hg were 50.24, 3.81, 2.64, 1.67, and 1.03 times the corresponding maximum limits of the national standards of China, respectively. Samples of pig-manure origin possessed higher average concentrations of Hg (0.25 mg/kg), As (9.10 mg/kg), and Pb (41.98 mg/kg), whereas samples of chicken-manure origin harbored higher average concentrations of Cd (1.37 mg/kg) and Cr (39.66 mg/kg). However, no samples from dairy feces and plant waste were identified to contain heavy metals exceeding the national standard. The concentrations of Cr and Pb in samples from chicken, pig, and dairy feces were significantly higher than those of plant waste origin (*P < *0.05; Fig. 1).

**TABLE 1:** Comparison of residual concentrations of heavy metals in fertilizer samples of different origins^*a*^^,^^*b*^*^,^*^*c*^

	Chicken feces origin (*n* = 34)			Pig feces origin (*n* = 27)			Dairy feces origin (*n* = 18)			Plant waste origin (*n* = 15)			Total (*n* = 94)		
Heavy metal	Concn range (mg/kg)	Mean (mg/kg)	Ratio (%)	Concn range (mg/kg)	Mean (mg/kg)	Ratio (%)	Concn range (mg/kg)	Mean (mg/kg)	Ratio (%)	Concn range (mg/kg)	Mean (mg/kg)	Ratio (%)	Concn range (mg/kg)	Mean (mg/kg)	Ratio (%)
Hg	ND–0.75	0.16	0	ND–2.07	**0.25**	3.70	ND–0.19	0.04	0	ND–0.61	0.12	0	ND–2.07	0.16	1.06
As	0.03–27.72	5.96	2.94	2.88–121.56	**12.13**	14.81	0.25–6.34	4.52	0	0.41–8.96	3.67	0	0.03–121.56	7.09	5.32
Cd	0.01–11.42	**1.37**	8.82	0.06–2.74	0.86	0	0.04–1.14	0.39	0	0.01–1.31	0.47	0	0.01–11.42	0.89	3.19
Cr	2.19–250	**39.66**	5.88	6.11–95.73	32.98	0	5.42–75.80	25.90	0	2.55–37.93	13.52	0	2.19–250	30.94	2.13
Pb	0.23–251.19	25.70	5.88	0.33–505.93	**41.98**	3.70	0.64–46.73	16.28	0	0.46–39.93	7.95	0	0.23–251.19	25.74	6.38

a Ratio (%) is the portion of samples in which the content of heavy metals exceeded the maximal limits of the national standard.

b ND, not detected using the detection methods in this study.

c The values in bold were the highest means when comparing samples of different origins.

Of the 18 tested antimicrobials, a total of 10 antimicrobials were detected in 38 (40.43%) samples, and the concentrations ranged from 1.93 μg/kg to 480.70 μg/kg (Table 2 and Table S6). Of these samples, azithromycin was most frequently detected (13/94, 13.83%), followed by oxytetracycline and ciprofloxacin (7/94, 7.45%), sulfaquinoxaline and danofloxacin (5/94, 5.32%), tilmicosin and chlortetracycline (4/94, 4.26%), doxycycline (2/94, 2.13%), and sulfadiazine and thiamphenicol (1/94, 1.06%). The residual concentrations of macrolides, tetracyclines, fluoroquinolones, sulfanilamides, and amphenicols were 1.93 to 8.93 μg/kg, 8.51 to 137.87 μg/kg, 4.99 to 480.70 μg/kg, 3.18 to 11.07 μg/kg and 44.46 μg/kg, respectively. There were six samples (6.38%) in which the concentration of at least one antimicrobial exceeded the ecotoxic effect trigger value of 100 μg/kg (23). As shown in Table 2, the detection ratios of sulfanilamides and macrolides were higher in fertilizer samples from chicken feces. In contrast, fluoroquinolones, tetracyclines, and amphenicols were more frequently detected in samples from pig feces. The total concentrations of five classes of antimicrobials were significantly higher in fertilizer samples from chicken, pig, and dairy feces than those of plant waste origin (*P < *0.01; Fig. S2).

**TABLE 2:** Comparison of residual concentrations of antimicrobials in fertilizer samples of different origins^*a*^

Antibiotic	Chicken feces origin (*n* = 34)			Pig feces origin (*n* = 27)			Dairy feces origin (*n* = 18)			Plant waste origin (*n* = 15)			Total (*n* = 94)		
	Detection rate (%)	Concn range (μg/kg)	Mean (μg/kg)	Detection rate (%)	Concn range (μg/kg)	Mean (μg/kg)	Detection rate (%)	Concn range (μg/kg)	Mean (μg/kg)	Detection rate (%)	Concn range (μg/kg)	Mean (μg/kg)	Detection rate (%)	Concn range (μg/kg)	Mean (μg/kg)
Sulfonamides	8.82	7.90–11.07	8.43	3.70	5.76	5.76	5.56	5.03	5.03	6.67	3.18	3.18	6.38	3.18–11.07	6.55
Sulfadiazine	0			0			5.56	5.03	5.03	0			1.06	5.03	5.03
Sulfamethoxazole	0			0			0			0			0		
Sulfamethazine	0			0			0			0			0		
Sulfamonomethoxine	0			0			0			0			0		
Sulfaquinoxaline	8.82	7.90–11.07	8.43	3.70	5.76	5.76	0			6.67	3.18	3.18	5.32	3.18–11.07	6.85
Fluoroquinolones	11.76	29.54–494.56	186.16	14.81	11.47–114.36	47.87	11.11	27.64–212.48	120.06	6.67	4.99	4.99	12.77	4.99–480.7	98.44
Enrofloxacin	0			0			0			0			0		
Ciprofloxacin	8.82	13.86–118.46	53.95	7.41	11.47–11.93	11.70	5.56	27.64	27.64	6.67	4.99	4.99	7.45	4.99–118.46	31.13
Danofloxacin	5.88	102.09–480.70	291.40	7.41	53.7–114.36	84.03	5.56	212.48	212.48	0			5.32	53.7–480.7	192.67
Sarafloxacin	0			0			0			0			0		
Macrolides	26.47	7.36–8.93	8.33	7.41	8.74–8.85	8.80	22.22	7.40–8.73	8.25	13.33	1.93–2.07	2	18.09	1.93–8.93	7.62
Azithromycin	20.59	8.36–8.93	8.55	7.41	8.74–8.85	8.80	16.67	8.38–8.73	8.54	6.67	1.93	1.93	13.83	1.93–8.93	8.08
Tilmicosin	5.88	7.36–7.72	7.54	0			5.56	7.40	7.40	6.67	2.07	2.07	4.26	2.07–7.72	6.14
Tylosin	0			0			0			0			0		
Tetracyclines	14.71	15.01–259.31	61.15	18.52	16.96–79.41	52.81	5.56	22.29	22.29	0			13.83	8.51–137.87	45.55
Tetracycline	0			0			0			0			0		
Oxytetracycline	8.82	15.01–137.87	56.21	11.11	16.96–44.2	28.09	5.56	13.78	13.78	0			7.45	13.78–137.87	38.1
Chlortetracycline	5.88	15.68–121.44	68.56	3.70	56.31	56.31	5.56	8.51	8.51	0			4.26	8.51–121.44	50.49
Doxycycline	0			7.41	59.32–64.16	61.74	0			0			2.13	59.32–64.16	61.74
Amphenicols	0			3.70	44.46	44.46	0			0			1.06	44.46	44.46
Thiamphenicol	0			3.70	44.46	44.46	0			0			1.06	44.46	44.46
Florfenicol	0			0			0			0			0		

a The mean is the average value of the detected concentrations of antimicrobials.

A total of 18 types of pesticide residue were detected in 48 (51.06%) samples, and the concentrations ranged from 2.16 μg/kg to 828.76 μg/kg (Table S7). Cyromazine was detected most frequently (27/94, 28.72%), followed by tricyclazole and dichlorvos (6/94, 6.38%), diethofencarb, tebuconazole, and isocarbophos (5/94, 5.32%), prochloraz, carbendazim, and imidacloprid (3/94, 3.19%), fenpropathrin, methyl parathion, chlorpyrifos, and avermectin (2/94, 2.13%), and triazophos, bifenthrin, carbaryl, tetramethrin, and 3-OH-carbofuran (1/94, 1.06%). The average detectable concentration for cyromazine was 82.29 μg/kg, and the average residual concentrations for the other pesticides were below 20 μg/kg. Cyromazine was more frequently detected (32.35% to 38.89%) in fertilizer samples from chicken, pig, and dairy feces. However, no cyromazine was detected in fertilizer samples of plant waste origin (Table S7). Other than cyromazine, the samples of plant waste origin contained a more variety and higher concentrations of pesticides than the fertilizer samples from chicken, pig, and dairy feces.

### Detection frequency and concentrations of *tet*(X)-variant genes.

**FIG 2:**
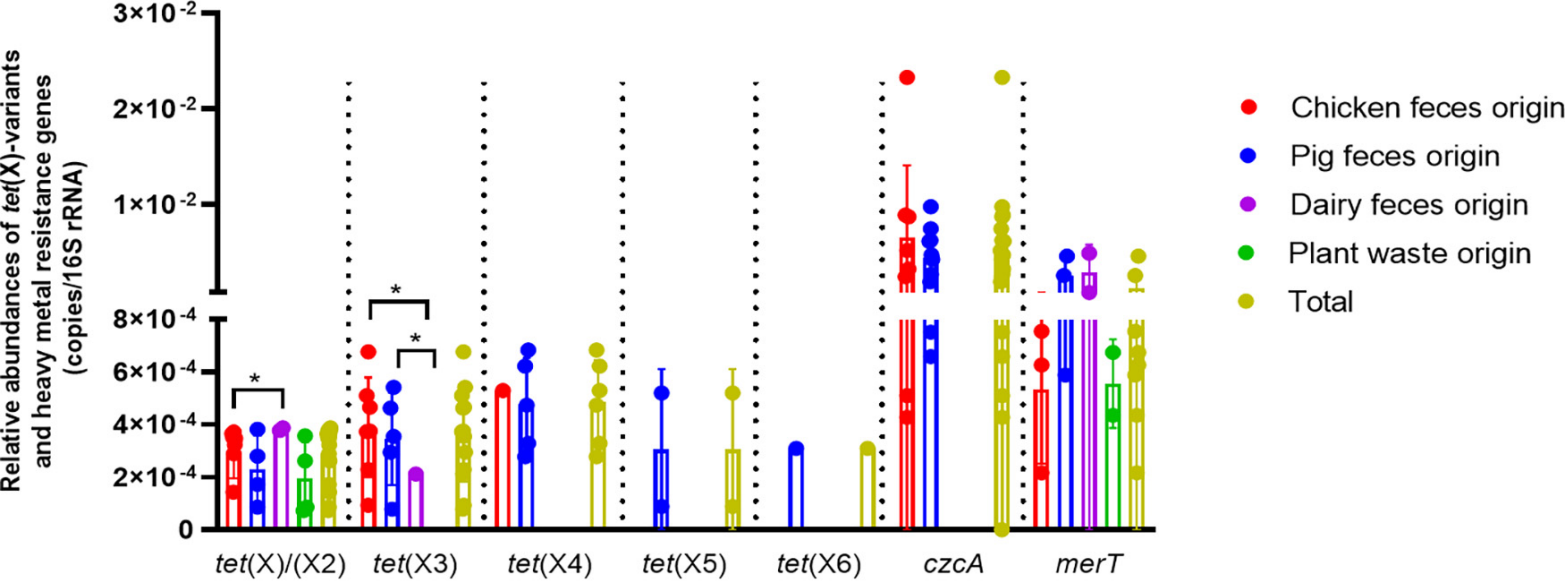
Relative abundances (copies/16S rRNA) of *tet*(X)-variant genes and heavy metal resistance genes (*czcA* and *merT*) in fertilizer samples of different origins. An unpaired *t test* was used to perform statistical significance; *, *P < *0.05, two-tailed.

Of the 94 DNA samples, *tet*(X)/(X2), *tet*(X3), *tet*(X4), *tet*(X5), and *tet*(X6) were detected in 34 (36.2%) samples (15 samples from chicken feces, 12 samples from pig feces, 3 samples from dairy feces, and 4 samples of plant waste origin [Table 3]). Among the *tet*(X)-variant-positive samples, *tet*(X)/(X2) was identified in 17 fertilizers (43.59%, 17/39), *tet*(X3) in 13 fertilizers (33.33%), *tet*(X4) in 6 fertilizers (15.38%), *tet*(X5) in 2 fertilizers (5.13%), and *tet*(X6) in 1 fertilizer (2.56%) (Table S8). The relative abundances were 7.36 × 10^−5^ to 3.88 × 10^−4^ copies/16S rRNA, 7.9 × 10^−5^ to 6.77 × 10^−4^ copies/16S rRNA, 2.78 × 10^−4^ to 6.84 × 10^−4^ copies/16S rRNA, 8.94 × 10^−5^ to 5.21 × 10^−4^ copies/16S rRNA, and 3.10 × 10^−4^ copies/16S rRNA for *tet*(X)/(X2), *tet*(X3), *tet*(X4), *tet*(X5), and *tet*(X6), respectively (Fig. 2). The average relative abundance of *tet*(X4) (4.86 × 10^−4^ copies/16S rRNA) was higher than that of *tet*(X)/(X2) (2.77 × 10^−4^ copies/16S rRNA) and *tet*(X3) (3.59 × 10^−4^ copies/16S rRNA). With respect to samples of different origins, *tet*(X)/(X2) was highly detected in fertilizers of plant waste origin (26.67%), whereas *tet*(X3) and *tet*(X4) were mostly detected in samples from chicken feces (20.59%) and pig feces (18.52%). Samples from pig feces harbored six kinds of *tet*(X)-variants, including *tet*(X)/(X2), *tet*(X3), *tet*(X4), *tet*(X5), and *tet*(X6), whereas samples of plant waste origin harbored only *tet*(X)/(X2) (Fig. 2).

**TABLE 3:** Comparison of relative abundances (copies/16S rRNA) of *tet*(X)-variants in fertilizer samples of different origins

Gene	Chicken feces origin (*n* = 34)				Pig feces origin (*n* = 27)				Dairy feces origin (*n* = 18)				Plant waste origin (*n* = 15)				Total (*n* = 94)			
	Detection ratio (%)	Minimum	Maximum	Mean	Detection ratio (%)	Minimum	Maximum	Mean	Detection ratio (%)	Minimum	Maximum	Mean	Detection ratio (%)	Minimum	Maximum	Mean	Detection ratio (%)	Minimum	Maximum	Mean
*tet*(X)/(X2)	23.53	1.43 × 10^−4^	3.72 × 10^−4^	2.92 × 10^−4^	11.1	1.73 × 10^−4^	3.82 × 10^−4^	2.79 × 10^−4^	11.11	3.78 × 10^−4^	3.88 × 10^−4^	3.83 × 10^−4^	26.67	7.36 × 10^−5^	3.57 × 10^−4^	1.95 × 10^−4^	18.09	7.36 × 10^−5^	3.88 × 10^−4^	2.77 × 10^−4^
*tet*(X3)	20.59	9.40 × 10^−5^	6.77 × 10^−4^	3.89 × 10^−4^	18.52	7.90 × 10^−5^	5.42 × 10^−4^	3.47 × 10^−4^	5.56	2.12 × 10^−4^	2.12 × 10^−4^	2.12 × 10^−4^	0				13.83	7.90 × 10^−5^	6.77 × 10^−4^	3.59 × 10^−4^
*tet*(X4)	2.94	5.30 × 10^−4^	5.30 × 10^−4^	5.30 × 10^−4^	18.52	2.78 × 10^−4^	6.84 × 10^−4^	4.78 × 10^−4^	0				0				6.38	2.78 × 10^−4^	6.84 × 10^−4^	4.86 × 10^−4^
*tet*(X5)	0				7.41	8.94 × 10^−5^	5.21 × 10^−4^	3.05 × 10^−4^	0				0				2.13	8.94 × 10^−5^	5.21 × 10^−4^	3.05 × 10^−4^
*tet*(X6)	0				3.70	3.10 × 10^−4^	3.10 × 10^−4^	3.10 × 10^−4^	0				0				1.06	3.10 × 10^−4^	3.10 × 10^−4^	3.10 × 10^−4^

Two kinds of heavy metal resistance genes (*czcA* and *merT*) were detected in 20 and 8 samples, respectively, and the relative abundances of *czcA* and *merT* were in the range of 4.28 × 10^−4^ to 2.33 × 10^−2^ copies/16S rRNA and 2.17 × 10^−4^ to 4.63 × 10^−3^ copies/16S rRNA, respectively (Table S8). Notably, 61.5% (8/13) of the *tet*(X3)-positive samples carried *czcA*, and 33.3% (2/6) of *tet*(X4)-positive samples harbored *merT.* The relative abundance of the *tet*(X3) gene showed a significantly positive correlation (*R* = 0.474, *P < *0.001) with that of *czcA*, and the relative abundance of the *tet*(X4) gene showed a significantly positive correlation (*R* = 0.266, *P < *0.01) with that of *merT* (Table S9). The relative abundance of three plasmid *rep* genes [belonging to *Enterobacteriaceae* IncX1, IncFIA(HI1), and IncFIB(K) plasmids, respectively] was significantly correlated with that of the *tet*(X4) gene (*R* = 0.231 to 0.318, *P < *0.05), whereas the relative abundance of *rep* genes from Acinetobacter plasmids pB18-2 and p34AB showed a significantly positive correlation with that of the *tet*(X3) gene (*R* = 0.221 to 0.264, *P < *0.05) (Table S9). However, there was no significant correlation between the relative abundance of any *rep* gene with that of the *tet*(X5) or *tet*(X6) gene.

### Microbial diversity and exogenous plasmid isolation.

All the *tet*(X)-variant-positive samples were used to isolate the tigecycline-resistant bacteria. However, no tigecycline-resistant bacteria were obtained from these samples (data not shown). The microbial diversity of these samples was analyzed. The phyla *Firmicutes* and *Actinobacteriota* were most abundant in both *tet*(X)-variant-positive and *tet*(X)-variant-negative samples (Fig. S3). For *tet*(X)-variant-positive samples, the *Proteobacteria* were more abundant in *tet*(X3)/*tet*(X4)-positive samples than in *tet*(X)/*tet*(X2)-positive samples. When the community composition was analyzed at the genus level, more than 1,000 bacterial genera were identified among both *tet*(X)-variant-positive and *tet*(X)-variant-negative samples. Although previous studies showed that Acinetobacter spp. and *Enterobacteriaceae* bacteria were the predominant bacterial hosts for *tet*(X3) and *tet*(X4) genes (24, 25), the genera, including Acinetobacter and Escherichia, were not more abundant in the *tet*(X3)/*tet*(X4)-positive samples than in the *tet*(X)-variant-negative samples (data not shown). To assess the transfer risk of *tet*(X)-variant genes, exogenous plasmid isolation from the *tet*(X4)-positive fertilizer samples was conducted. Of the six *tet*(X4)-positive samples, *tet*(X4)-carrying transformants were identified in three samples (YJF-2020014, YJF-2020016, and YJF-2020079) (Table S8). Nine *tet*(X4)-positive transformants (three for each sample) were submitted for whole-genome sequencing (WGS), and the *tet*(X4) gene was located on the plasmids, indicating that the *tet*(X4)-carrying plasmids from these samples were captured by exogenous E. coli. For each sample, only one kind of *tet*(X4)-bearing plasmid was identified by WGS, and two samples (YJF-2020014 and YJF-2020016) harbored a similar plasmid. Therefore, two kinds of *tet*(X4)-bearing plasmids (pRF14-1-like and pYPE10-like) were identified among the three samples, which showed >99% coverage and >99% sequence identity with the previously reported conjugative *tet*(X4)-bearing plasmids pRF14-1 (GenBank accession no. NZ_MT219822) and pYPE10 (GenBank accession no. NZ_CP041449), respectively (Fig. S4) (26). The plasmid stability assay showed that all the selected clones in each passage were positive for the *tet*(X4) gene, indicating that the pRF14-1-like and pYPE10-like plasmids were stable in exogenous E. coli.

### Correlations between *tet*(X)-variants and environmental variables.

**FIG 3:**
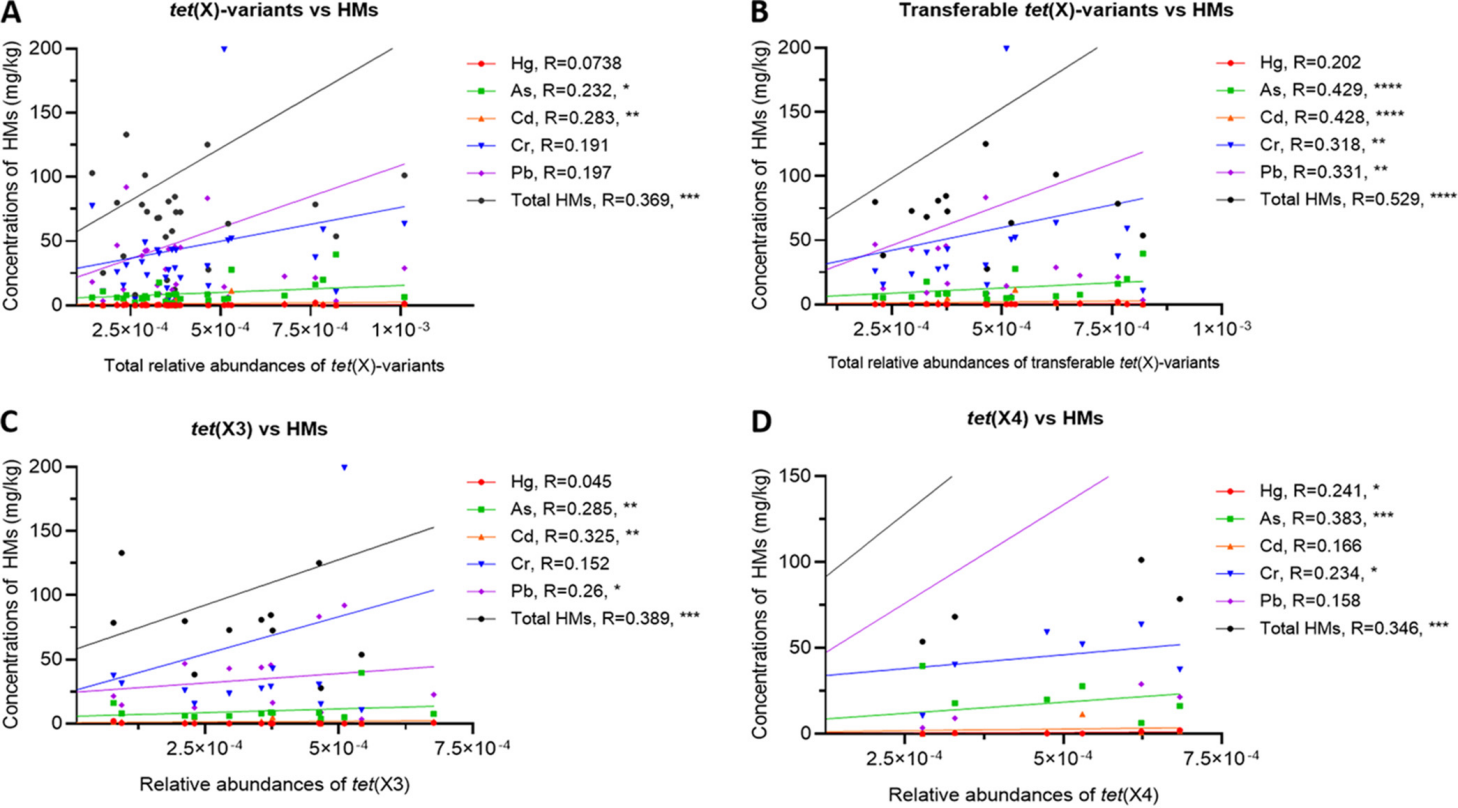
Correlation analysis between the relative abundances of *tet*(X)-variant genes (copies/16S rRNA) and the residual concentrations (mg/kg) of heavy metals (HMs) by Spearman correlation analysis. (A) The total relative abundance of *tet*(X)-variant genes [*tet*(X)/(X2), *tet*(X3), *tet*(X4), *tet*(X5), and *tet*(X6)] versus concentrations of different heavy metals. (B) The total relative abundance of the transferable *tet*(X)-variant genes [*tet*(X3), *tet*(X4), *tet*(X5), and *tet*(X6)] versus concentrations of different heavy metals. (C) The relative abundance of *tet*(X3) versus concentrations of different heavy metals. (D) The relative abundance of the *tet*(X4) versus concentrations of different heavy metals. Linear regression analysis was performed with GraphPad Prism v8.0. *R*, the Spearman correlation coefficient. *, *P < *0.05, two-tailed; **, *P < *0.01, two-tailed; ***, *P < *0.001, two-tailed; ****, *P < *0.0001, two-tailed.

Redundancy analysis (RDA) was performed to assess the potential relationships between the distribution of *tet*(X) variant genes and various environmental variables. All the selected variables (five classes of antimicrobials, five heavy metals, and all pesticides) explained 43.3% of the distribution variation of *tet*(X)-variants. Of these variables, the five heavy metals and five classes of antimicrobials contributed 87.8% and 11% of the variation, respectively (Fig. S5). The total relative abundance of the *tet*(X)-variant genes [∑*tet*(X)-variants] showed a significantly positive correlation with the total concentrations of heavy metals (Fig. 3A; *R* = 0.369, *P < *0.001). With respect to different *tet*(X) variants, *tet*(X)/(X2) showed no significant correlation with any of the five heavy metals. In contrast, the total relative abundance of the transferable *tet*(X)-variant genes, including *tet*(X3), *tet*(X4), *tet*(X5), and *tet*(X6), was significantly correlated with the total concentration of heavy metals (Fig. 3B; *R* = 0.529, *P < *0.0001). The *tet*(X3)-variant showed significant correlations with As (*R* = 0.285, *P < *0.01), Cd (*R* = 0.325, *P < *0.01), and Pb (*R* = 0.26, *P < *0.05) (Fig. 3C). The *tet*(X4)-variant showed significantly positive correlations with Hg (*R* = 0.241, *P < *0.05), As (*R* = 0.383, *P < *0.001), and Cr (*R* = 0.234, *P < *0.05) (Fig. 3D). However, *tet*(X5) and *tet*(X6) showed no significant correlations with any of the heavy metals.

For antimicrobials, Spearman correlation analysis revealed that the total relative abundance of the detected *tet*(X)-variant genes [∑*tet*(X)-variants] showed no significant correlation with any of the 18 antimicrobials. However, the relative abundance of *tet*(X4) showed a significant correlation with the concentrations of oxytetracycline (*R* = 0.275, *P < *0.01) and doxycycline (*R* = 0.257, *P < *0.05), while the relative abundance of *tet*(X6) showed a significant correlation with the concentration of doxycycline (*R* = 0.696, *P < *0.0001) (Table S9), suggesting direct selection of the *tet*(X)-variants by antimicrobials of the tetracycline class. For the pesticides, there was no significant correlation between the relative abundance of *tet*(X)-variants and the concentrations of the pesticides.

### Impacts of heavy metals on conjugation.

**FIG 4:**
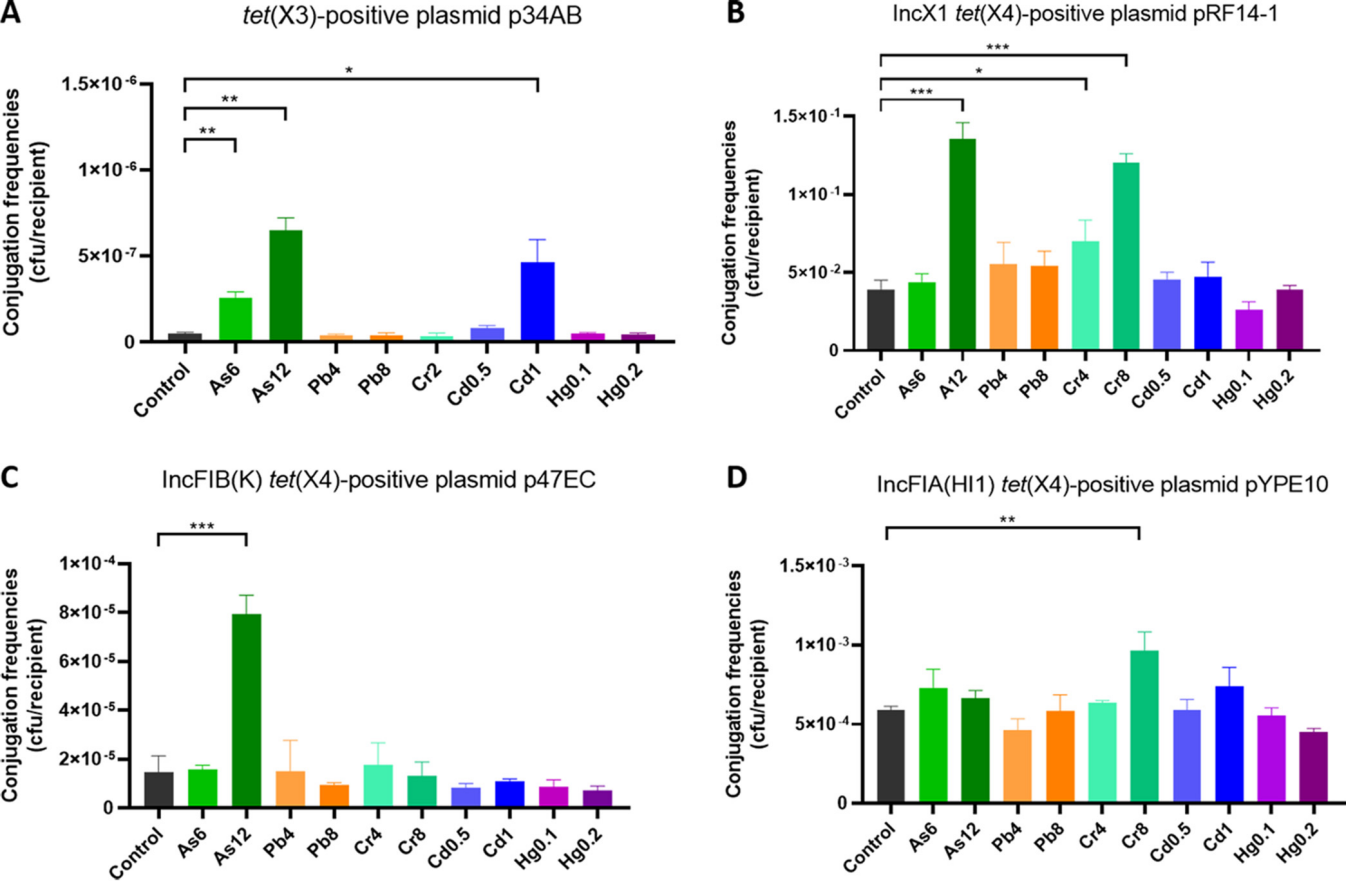
Effect of heavy metals on the conjugation efficiencies of *tet*(X3)- and *tet*(X4)-carrying plasmids. (A) Conjugation efficiencies of *tet*(X3)-carrying plasmid p34AB from A. baumannii ATCC 17978 to *bla*_OXA-23_-positive A. baumannii 5AB. (B) Conjugation efficiencies of InX1 *tet*(X4)-carrying plasmid pRF14-1 from E. coli BL21 to E. coli J53. (C) Conjugation efficiencies of IncFIB(K) *tet*(X4)-carrying plasmid p47EC from E. coli BL21 to E. coli J53. (D) Conjugation efficiencies of IncFIA(HI1) *tet*(X4)-carrying plasmid pYPE10 from E. coli BL21 to E. coli J53. Each test was performed with three independent repeats, and the error bars show the standard deviations (SDs). The significant difference between conjugation efficiencies of the plasmid under one heavy metal and the corresponding control was tested using the unpaired *t* test; *, *P < *0.05; **, *P < *0.01; ***, *P < *0.001.

As shown in Fig. 4A, As (6 μg/mL and 12 μg/mL) and Cd (1 μg/mL) significantly promoted the conjugation efficiency of *tet*(X3)-carrying plasmid p34AB from Acinetobacter baumannii ATCC17978 to an A. baumannii 5AB recipient, with conjugation frequencies of 2.2 × 10^−7^ to 7.3 × 10^−7^, compared with the controls of 4 × 10^−8^ to 5.79 × 10^−8^ (*P < *0.05). There was no significant difference in conjugation frequencies of plasmid p34AB between the controls and that with the selective pressure of the other heavy metals. For *tet*(X4), As (12 μg/mL) and Cr (8 μg/mL) significantly promoted the conjugation efficiency of IncX1-plasmid pRF14-1 from E. coli BL21 to the E. coli J53 recipient (Fig. 4B; *P < *0.001). In addition, As (12 μg/mL) significantly promoted the conjugation efficiency of IncFIB(K)-plasmid p47EC (Fig. 4C; *P < *0.001), and Cr (8 μg/mL) significantly promoted the conjugation efficiency of IncFIA(HI1)-plasmid pYPE10 (Fig. 4D; *P < *0.01). There was no significant difference in conjugation frequencies of the selected *tet*(X4)-carrying plasmids between the bacteria with the selective pressure of other heavy metals and the corresponding controls.

### Effects of heavy metals on the penetration of the cell membrane.

The effect of heavy metals on the penetration of the cell membrane was evaluated using an outer membrane permeability assay. After exposure to the subinhibitory concentrations of different heavy metals, the fluorescence of 1-N-phenylnaphthylamine (NPN)-probed cells was enhanced by less than 2-fold in the donor strains carrying the corresponding *tet*(X3)/*tet*(X4)-positive plasmid (Fig. S6). Furthermore, the fluorescence intensity was increased when the concentrations of heavy metals increased, indicating that the heavy metals disrupted the integrity of the bacterial outer membrane and increased the penetration of transferable genetic elements, such as plasmids, across the cell membrane.

### Effects of heavy metals on the expression of T4SS-encoding genes.

**FIG 5:**
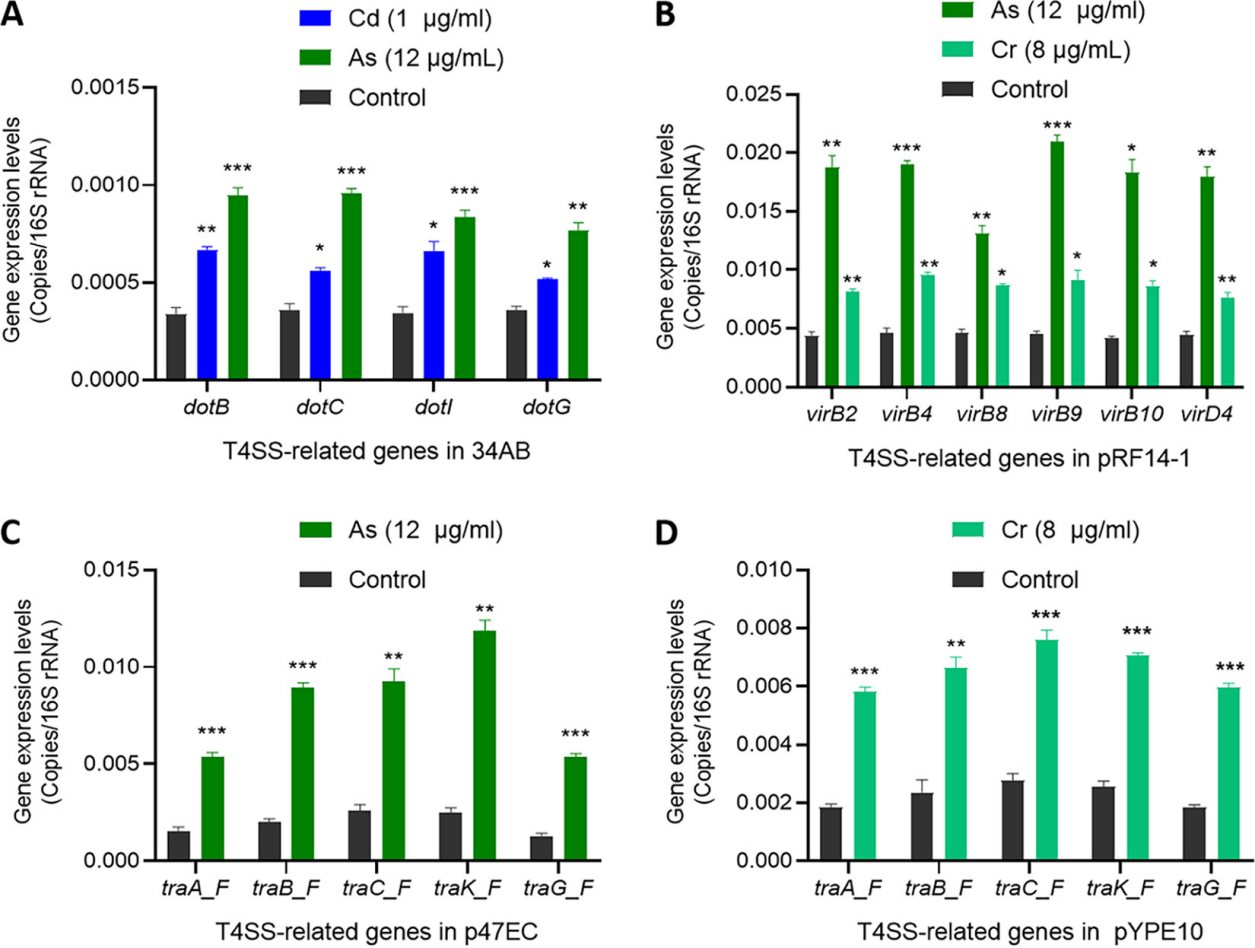
Effect of heavy metals on the expression levels of T4SS-related genes on *tet*(X3)/*tet*(X4)-carrying plasmids. (A) Transcriptional levels of T4SS-related genes on *tet*(X3)-carrying plasmid p34AB. (B) Transcriptional levels of T4SS-related genes on *tet*(X4)-carrying plasmid pRF14-1. (C) Transcriptional levels of T4SS-related genes on *tet*(X4)-carrying plasmid p47EC. (D) Transcriptional levels of T4SS-related genes on *tet*(X4)-carrying plasmid pYPE10. Each test was performed with three independent repeats, and the error bars show the standard deviations (SDs). The significant difference between transcriptional levels of T4SS-related genes under one heavy metal and the corresponding control was tested using the unpaired *t test*; *, *P < *0.05; **, *P < *0.01; ***, *P < *0.001.

Quantitative PCR (qPCR) was performed to analyze the changes in the expression of T4SS-related genes in different plasmids (Fig. 5). The transcriptional levels of *dotB*, *dotC*, *dotI*, and *dotG* genes in the *tet*(X3)-carrying plasmid p34AB were significantly increased by 2- to 3-fold (Fig. 5A; *P < *0.05) under the selection pressure of As (12 μg/mL) and Cd (1 μg/mL). For *tet*(X4)-carrying plasmids, the expression levels of five selected genes (type-F T4SS: *traA_F*, *traB_F*, *traC_F*, *traK_F*, and *traG_F*) in plasmid p47EC and six genes (type-P T4SS: *virB2*, *virB4*, *virB8*, *virB9*, *virB10*, and *virD4*) in plasmid pRF14-1 were significantly upregulated by 3- to 4-fold (*P < *0.01) under the selection pressure of As (12 μg/mL), whereas the expression levels of six genes (*virB2*, *virB4*, *virB8*, *virB9*, *virB10*, and *virD4*) in pRF14-1 and five genes in pYPE10 (type-F T4SS: *traA_F*, *traB_F*, *traC_F*, *traK_F*, and *traG_F*) were significantly upregulated by 2- to 3-fold with Cr (8 μg/mL) (Fig. 5). The significant upregulation of T4SS-encoding genes was consistent with the increased transferring frequencies of these plasmids in conjugation assays. However, there was no significant difference between the transcriptional levels of the T4SS-encoding genes with the selection of other heavy metals (Hg and Pb) and those without these heavy metals.

## DISCUSSION

With the increased global consumer demand for organic food, the use of commercial organic fertilizers is increasing, especially in China (22). Although commercial organic fertilizers are considered safer than animal manures or animal feces compost, heavy metals, veterinary antimicrobials, and pesticides contaminated commercial organic fertilizers, with the heavy metals being detected most frequently among the collected samples. Apart from chemical pollutants, tigecycline-resistant *tet*(X)-variant genes were detected, and both veterinary antimicrobials and heavy metals showed significantly positive correlations with the relative abundance of the *tet*(X)-variant genes, which suggests that these two chemical contaminants may act as the potential drivers for the transmission of the *tet*(X)-variant genes.

The *tet*(X)-variant genes conferred resistance to all antimicrobials of the tetracycline class (4). Therefore, the veterinary tetracyclines, especially oxytetracycline, chlortetracycline, and doxycycline, which were extensively used as growth promoters in animal feed before 2020, could serve as a direct driver for the transmission of *tet*(X)-variant genes. The coselection of the *tet*(X)-variants by other antimicrobial classes (e.g., quinolones and macrolides) was present, as the coexistence of multiple resistance genes, such as *tet*(X3) with *sul2*, *mph*(E), and *msr*(E) or *tet*(X4) with *floR*, *qnrS1*, *oqxAB*, and *mph*(A) in one plasmid has been identified in previous reports (15, 25). Although China has issued an antimicrobial-additive ban on animal feed, the selective effect of veterinary antimicrobials on the *tet*(X)-variant genes should not be neglected, as the tetracyclines are still the predominant antimicrobial class used in clinical treatment for animal diseases (27).

Due to the antimicrobial ban, heavy metals may play a more important role in spreading the *tet*(X)-variant genes, as the metals inevitably contaminated animal feces and were present in animal feces-derived fertilizers (28, 29). Heavy metals are one kind of persistent pollutant and could serve as a consistent selective pressure for the spread of *tet*(X)-variant genes, as a coselection effect was confirmed between *tet*(X)-variant genes and heavy metal resistance genes in this study. We conducted a comparative analysis of the published *tet*(X3)-carrying and *tet*(X4)-carrying plasmids obtained from NCBI, and *tet*(X3) frequently coexisted with Cr resistance gene *czc*A. *tet*(X4) was more likely to coexist with Hg resistance gene *merT* in the same plasmid (Table S10). As intensive animal production systems are still increasing in China, a large amount of animal manure will be produced and made into organic fertilizers each year (30). The sustained application of organic fertilizers in farmlands causes the accumulation of metals in soil that may create a consistent selective pressure for the spread of *tet*(X)-variant genes within the environment in the future (31).

The plasmids play a significant role in the horizontal transfer of *tet*(X3) and *tet*(X4) genes (4, 5, 25). In this study, *tet*(X3) and *tet*(X4) were highly detected among all the transferable *tet*(X) variants [*tet*(X3) to *tet*(X6)] and were significantly correlated with the relative abundance of several plasmid *rep* genes, implying the possible location of *tet*(X3)/*tet*(X4) genes on the plasmids of the corresponding incompatible groups [e.g., IncX1, IncFIA(HI1), and IncFIB(K)]. As commercial organic fertilizers have a strict microbial limit (<100/g) according to Chinese fertilizer standard NY525-2012, the *tet*(X3)/*tet*(X4)-carrying bacteria were not isolated from these fertilizer samples. The *tet*(X4)-carrying plasmids [IncX1 and IncFIA(HI1)] from some fertilizers can be captured by exogenous E. coli and were stably present in the recipient strain, indicating a high transmission risk of the *tet*(X)-variant gene within the environment if the fertilizers were applied in the farmland.

In addition to coselection, the heavy metal could promote the conjugation ability of the *tet*(X3)/*tet*(X4)-carrying plasmids. The heavy metal concentrations used in conjugation assays were lower than the maximum residual limits in NY 525-2012, which indicated that the transmission risk of *tet*(X)-variant genes might be underestimated, as low concentrations of heavy metals were widely detected in organic fertilizers and the animal manure-related environment (32, 33). The underlying mechanism of the conjugation-promoting effect was that heavy metals increased the permeability of the bacterial outer membrane and upregulated the transcriptional levels of T4SS-encoding genes on the corresponding *tet*(X)-variant-carrying plasmids. The T4SSs are multisubunit cell-envelope-spanning structures composed of a conjugative pilus, outer membrane core complex, inner membrane complex, and energy center complex (34). Our findings indicated that the related T4SS-component genes were upregulated by heavy metals, including the pilus-encoding gene (P-type: *virB2*; F-type homolog: *traA*), outer membrane complex-encoding genes (P-type: *virB9* and *virB10*; F-type homolog: *traK* and *traB*), inner membrane complex-encoding genes (P-type: *virB8*; F-type: *traG*), and energy center-encoding genes (P-type: *virB4*; F-type homolog: *traC*). Previous studies indicated that the conjugation of IncP-plasmid RP4 could be enhanced by nanoalumina and subinhibitory concentrations of colistin, which is due to the upregulation of conjugative regulatory genes (*trfAp* and *trbBp*) on RP4 promoted by these chemicals (35, 36). However, RP4 is only one model plasmid carrying the type-P T4SS, and there are many other T4SSs (e.g., type F and type I) identified in the clinically resistant plasmids (37). According to our study, the T4SSs could be used as an indicator of the transferability of wild-type plasmids. The upregulated transcriptional level of T4SS may increase the mating-pair formation between donor and recipient strains, which increases the transfer frequencies of the resistant plasmids (38).

**Conclusion.** This study showed that heavy metals, other than veterinary antimicrobials, may play an important role in spreading *tet*(X)-variant genes within the animal manure-related environment, especially the transferable *tet*(X3) and *tet*(X4) genes. Heavy metals coselected *tet*(X3)/*tet*(X4) genes and promoted the conjugation ability of the *tet*(X3)/*tet*(X4)-carrying plasmids. Due to the antimicrobial additive ban in China, the potential role of heavy metals in the spread of *tet*(X)-variant genes within the animal manure-related environment should not be neglected, and consistent monitoring is needed.

## MATERIALS AND METHODS

### Sample collection.

Samples of commercial organic fertilizers were collected in nine representative provinces, which were located in Northeast (Heilongjiang and Liaoning), North (Hebei), East (Shandong, Jiangsu, and Zhejiang), Central (Henan), South (Hubei), and Southwest (Sichuan) China during September to October in 2020 (Fig. S1). In each province, several typical organic fertilizer-producing factories were selected. At each factory, 10 subsamples were collected from different product batches and mixed thoroughly into a composite sample (~500 g). A total of 94 samples were obtained, including 18 samples from Hebei, 26 samples from Jiangsu, 16 samples from Zhejiang, 4 samples from Shandong, 5 samples from Henan, 8 samples from Hubei, 4 samples from Liaoning, 3 samples from Heilongjiang, and 10 samples from Sichuan (Table S1 and Fig. S1). The sources of raw materials for these fertilizers were investigated, and the labels of these products were recorded. These samples were sorted into four types: 34 fertilizers originating from chicken feces, 27 fertilizers from pig feces, 18 fertilizers from cattle feces, and 15 fertilizers from plant waste (straw from wheat, corn, rice, etc.). All the samples were transported to the laboratory on dry ice and then stored at −20°C before detection of the antimicrobials, heavy metals, pesticides, and *tet*(X)-variant genes.

### Quantification of heavy metal, antimicrobial, and pesticide residues.

Five heavy metals [mercury (Hg), arsenic (As), cadmium (Cd), chromium (Cr) and lead (Pb)], whose maximum residual limits in fertilizers are defined in the Chinese national standard NY 525-2012, were analyzed in 94 samples according to previously described methods (39). Briefly, the samples were digested in HNO_3_-HClO_4_ and analyzed by inductively coupled plasma spectrophotometry (ICP; IRIS-Advantage, Thermo, USA) for Pb, Cr, and Cd. The samples were digested in HNO_3_-H_2_SO_4_-HClO_4_ and examined by hydrogen atomic fluorescence spectroscopy for Hg and As (ASF-8230, Beijing Jitian, China).

A total of 18 antimicrobials belonging to 5 classes [sulfanilamides (SAs): sulfadiazine, sulfamethoxazole, sulfamethazine, sulfamonomethoxine, and sulfaquinoxaline; fluoroquinolones (FQs): enrofloxacin, ciprofloxacin, danofloxacin, and sarafloxacin; macrolides (MAs): azithromycin, tilmicosin, and tylosin; tetracyclines (TCs): oxytetracycline, chlortetracycline, tetracycline, and doxycycline; amphenicols (AMs): florfenicol and thiamphenicol], which are extensively used in animal husbandry in China, were analyzed among the samples. The analytical procedures for the target antimicrobials in these samples followed our previously described methods (15).

A total of 50 pesticides widely used in farmland were detected among these samples according to the protocols in NY 525-2012. Briefly, 5-g samples were homogenized in 50-mL centrifuge tubes and extracted ultrasonically for 10 min after adding 5 mL sterile water and 10 mL ethyl acetate (1%, pH 4.7). Then, 3 g of sodium chloride was added, and the mixtures were centrifuged for 10 min at 4,500 rpm. The supernatant was collected and analyzed by ultra-high-performance liquid chromatography and tandem mass spectrometry (UPLC+AB SCIEX 3500, Waters, USA). The 50 pesticides included 15 kinds of organophosphate insecticides (isocarbophos, malathion, dichlorvos, parathion-methyl, methamidophos, parathion, chlorophos, triazophos, profenofos, chlorpyrifos, phoxim, acephate, omethoate, diazinon, and dimethoate), 2 kinds of triazole fungicides (tebuconazole and difenoconazole), 4 kinds of carbamate insecticides (methomyl, carbofuran, 3-hydroxy-carbofuran, and carbaryl), 4 kinds of pyrethroids (fenpropathrin, tetramethrin, bifenthrin, and tau-fluvalinate), 3 kinds of nicotinamide insecticides (acetamiprid, imidacloprid, and thiamethoxam), 2 kinds of imidazole insecticides (imazalil and prochloraz), 5 kinds of mieyouniao insecticides (chlorbenzuron, chlorfluazuron, diflubenzuron, iprodione, and buprofezin), 4 kinds of phenyl pyrazole derivatives (fipronil, fipronil-desulfinyl, fipronil-sulfide, and fipronil-sulfone), and others (chlorantraniliprole, pyridaben, pyrimethanil, tricyclazole, dimethomorph, triadimefon, carbendazim, diethofencarb, cyromazine, avermectin B1a, and emamectin benzoate).

### DNA extraction and quantitative PCR (qPCR).

Total DNA was extracted using the PowerSoil DNA isolation kit (MoBio Laboratories, Carlsbad, CA, USA) according to the manufacturer’s instructions. qPCR was used to quantify the *tet*(X)-variant genes [*tet*(X) and *tet*(X2) to *tet*(X6)] and two heavy metal resistance genes, *czcA* (Cr resistance) and *merT* (Hg resistance), among all the DNA samples. As *tet*(X2) showed >99% nucleotide identity with the original, *tet*(X), *tet*(X)/*tet*(X2) was detected in total. The replicon-encoding (*rep*) genes in previously identified typical *tet*(X)-variant-carrying plasmids were also quantified, including *rep* genes from *Enterobacteriaceae tet*(X4)-carrying plasmids belonging to incompatible groups IncX1, IncFIA(HI1), IncFII, IncI1, and IncFIB(K) and those in representative *tet*(X3)/*tet*(X5)/*tet*(X6)-carrying plasmids (Table S2). All qPCR assays were performed on the LightCycler 480 real-time PCR system (Roche, Indianapolis, IN, USA) using TB green premix *Ex Taq* (TaKaRa, Tokyo, Japan). The primer pairs for qPCR are listed in Table S3. The *R*^2^ of linearity of the standard curve was >0.98, and the amplification efficiency ranged from 90% to 110%. Three replicates were included for each sample, and nuclease-free water was used as the negative control. The quantified concentrations of *tet*(X)-variant genes and heavy metal resistance genes were presented as relative abundance (copies/16S rRNA).

### Microbial diversity analysis and exogenous plasmid isolation.

All the *tet*(X)-variant-positive fertilizer samples were submitted for the analysis of microbial diversity. For comparison, several *tet*(X)-negative samples were selected for analysis, and each included two samples originating from pig feces, chicken feces, cow feces, and plant waste. Total DNA was extracted using the PowerSoil DNA isolation kit (MoBio Laboratories, Carlsbad, CA, USA). High-throughput sequencing was conducted on an Illumina NovaSeq PE250 platform at Shanghai Meiji Biotechnology Co. Ltd., Shanghai, China. Sequence analysis was performed with USEARCH software (v8.0.1517, http://www.drive5.com/usearch/). As the *tet*(X4)-positive samples were found to harbor plasmid *rep* genes belonging to diverse incompatibility groups, exogenous isolation of *tet*(X4)-carrying plasmids from the *tet*(X4)-positive samples was conducted. Briefly, the total DNAs of the *tet*(X4)-positive samples were introduced into Escherichia coli C600 competent cells by electrotransformation, with 2,500 V in a 2-mm electric rotor. The transformants were selected on Trypticase soy agar (TSA) supplemented with 2 μg/mL tigecycline, 50 μg/mL rifampicin, and 1,000 μg/mL streptomycin. The *tet*(X4)-positive transconjugants were identified by PCR. For each sample, three *tet*(X4)-positive clones were selected for whole-genome sequencing (WGS) using the Nanopore MinION and Illumina HiSeq platforms and were analyzed for their genetic contexts as described previously (26). The identified *tet*(X4)-carrying plasmids were then assessed for their stability in E. coli C600. After overnight growth of *tet*(X4)-positive transformants on Luria-Bertani (LB) agar, one clone from each strain was chosen randomly and incubated in tigecycline-free LB broth for 90 passages. In each passage, an aliquot of the grown culture was streaked on the LB agar, and 30 clones were selected for PCR to confirm the presence of the *tet*(X4) gene.

### Correlation analysis.

Statistical analysis was done using Prism v8.2.1 (GraphPad Software, La Jolla, CA, USA), and an unpaired *t* test was used to determine statistical significance based on *P* values (significance threshold, 0.05). Redundancy analysis (RDA) was performed using CANOCO v5, with the concentrations of *tet*(X)-variant genes as species and the concentrations of heavy metals/antimicrobials/pesticides as environmental variables. Spearman correlation was conducted to identify the association between the relative abundance of *tet*(X)-variant genes and residue levels of heavy metals, antimicrobials, or pesticides and between the relative abundance of *tet*(X)-variant genes and that of plasmid *rep* genes or the heavy metal resistance genes.

### Conjugation under the selective pressure of heavy metals.

To assess the effect of heavy metal on the transfer of the *tet*(X3)/*tet*(X4) gene, a conjugation assay was conducted by the filter mating method with/without subinhibitory concentrations of five heavy metals (Hg, Pb, Cr, As, and Cd) at 30°C for 12 h. The typical conjugative *tet*(X3)/*tet*(X4)-carrying plasmids were selected for conjugation, and *rep* genes have shown a significant positive correlation with the relative abundance of *tet*(X3)/*tet*(X4) as indicated in this study. For *tet*(X3), Acinetobacter baumannii ATCC 17978 harboring the *tet*(X3)-carrying plasmid p34AB (GenBank accession no. MK134375) was used as the donor, and the carbapenem-resistant clinical strain A. baumannii 5AB (*bla*_OXA-23_^+^) was used as the recipient strain. For *tet*(X4), Escherichia coli BL21 harboring the typical *tet*(X4)-carrying plasmids [IncX1 plasmid pRF14-1 (GenBank accession no. NZ_MT219822), IncFIB(K) plasmid p47EC (GenBank accession no. NZ_MK134376), and IncFIA(HI1) plasmid pYPE10 (GenBank accession no. NZ_CP041449), respectively] was used as the donor, and sodium azide-resistant E. coli J53 was used as the recipient (4, 26). Considering the lower bio-availability of heavy metals for bacteria, we proposed an average of 10% of each heavy metal as water-soluble that may be bio-available for bacteria, based on a previous study (40). Combining the bio-availability of heavy metals for bacteria and the MICs of heavy metals against the bacterial hosts (Table S4), the supplemented concentrations should be less than 10% of the maximum detected levels in fertilizers and have no effect on the normal growth of the bacteria. HgCl_2_, Pb(NO_3_)_2_, K_2_Cr_2_O_7_, Na_3_AsO_4_, and CdCl_2_ were used for MIC determination and conjugation assays (41). The heavy metal salts were dissolved in purified water and supplemented with specific concentrations in the LB agar for conjugation. The supplemented heavy metal concentrations were as follows: Hg^II+^ (0.1 μg/mL and 0.2 μg/mL), Pb^2+^ (4 μg/mL and 8 μg/mL), Cr^6+^ (2 μg/mL, or 4 μg/mL and 8 μg/mL), As^5+^ (6 μg/mL and 12 μg/mL), and Cd^2+^ (0.5 μg/mL and 1 μg/mL). Conjugation frequencies were calculated as the number of transconjugants/total number of recipients and compared between assays with the selective pressure of heavy metals and those without heavy metals. Each test was performed with three independent repeats. The unpaired *t* test was done to perform statistical significance based on *P* values (significance threshold, 0.05).

### Outer membrane permeability assay.

The A. baumannii ATCC 17978 carrying the *tet*(X3)-positive plasmid p34AB and E. coli BL21 carrying the *tet*(X4)-positive plasmid pRF14-1 (10^6^ CFU/mL) were treated with different concentrations of heavy metals as set in conjugation assays for 12 h. The cells were washed twice using phosphate-buffered saline (PBS), followed by fluorescent probe 1-N-phenylnaphthylamine (NPN) (10 mM) addition to evaluate the outer membrane integrity. After shaking at 100 rpm for 30 min in the dark, the fluorescence intensity was measured using a microplate system (Biotek Synergy v2, USA) at an excitation wavelength of 350 nm and an emission wavelength of 420 nm. Each test was performed with three independent repeats.

### mRNA (mRNA) expression of conjugative transfer-related genes.

As the type IV secretion system (T4SS) played a significant role in the conjugative transfer of the *tet*(X)-variant-carrying plasmids (Table S2), the transcriptional levels of T4SS-related genes on the above-described *tet*(X3)/*tet*(X4)-carrying plasmids in donor bacteria were investigated when mixed with the corresponding recipient strain at 30°C after 12 h with/without the selection of heavy metals. The T4SSs in the *tet*(X3)/*tet*(X4)-carrying-plasmids were predicted using the online SecReT4 tool and classified according to the T4SS classification system proposed by Lawley et al. (37) (https://bioinfo-mml.sjtu.edu.cn/SecReT4/t4ss_prediction.php). qPCR primers for the detection of T4SS component-encoding genes were designated using Primer3Plus (https://www.primer3plus.com/index.html). The bacterial RNA extract kit (Tiangen, Beijing, China) was used to extract the total RNA of the bacterial mixture after 12 h of conjugation. The extracted RNA was reverse-transcribed into cDNA using a PrimeScript RT reagent kit (TaKaRa, Tokyo, Japan). Real-time qPCR was used to quantify gene expression using TB green premix *Ex Taq* (TaKaRa, Tokyo, Japan). The transcriptional levels of typical T4SS-encoding genes in different plasmids were determined, including *dotB*, *dotC*, *dotI*, and *dotG* in plasmid p34AB (untypeable T4SS), *traA_F*, *traB_F*, *traC_F*, *traK_F*, and *traG_F* in plasmids p47EC and pYPE10 (both are type-F T4SS), and *virB2*, *virB4*, *virB8*, *virB9*, *virB10*, and *virD4* in plasmid pRF14-1 (type-P T4SS). 16S rRNA was used as the internal control. Primer sequences are listed in Table S3. The qPCR primers were synthesized by Qinke Biotech Co., Ltd. (Nanjing, China), and qPCR was performed on a LightCycler 480 real-time PCR system (Roche, Indianapolis, IN, USA). Three replicates were included for each sample, and nuclease-free water was used as the negative control.

### Data availability.

The 16S rRNA sequencing data in this study can be obtained from the NCBI under accession no. PRJNA923829. The two *tet*(X4)-carrying plasmids are available from the NCBI under accession no. OQ349371 (pRF14-1-like) and OQ322597 (pYPE10-like-1).
