# Supplementary material for: Comprehensive Analysis of Antimicrobial, Heavy Metal, and Pesticide Residues in Commercial Organic Fertilizers and Their Correlation with Tigecycline-Resistant tet(X)-Variant Genes
Source: Microbiol Spectr. 2023 Mar 14;11(2):e04251-22. doi: 10.1128/spectrum.04251-22 (PMC10100909; doi:10.1128/spectrum.04251-22)
Supplement: Supplemental file 1 — Supplemental material. Download spectrum.04251-22-s0001.pdf, PDF file, 1.0 MB [file spectrum.04251-22-s0001.pdf]

Comprehensive analysis of antimicrobial, heavy metal and pesticide residues in commercial organic fertilizers and their correlation with tigecycline-resistant *tet*(X)-variant genes

Tao He [a,*], Jun Li [a,*], Lan Gong [a], Yang Wang [b], Ruichao Li [c], Xing Ji [a], Fengting Luan [a], Minmin Tang [a], Lei Zhu [a], Ruicheng Wei [a,#], Ran Wang [a,#]

[a]Jiangsu Key Laboratory for Food Quality and Safety—State Key Laboratory Cultivation Base of Ministry of Science and Technology, Institute of Food Safety and Nutrition, Jiangsu Academy of Agricultural Sciences, Nanjing 210040, China
[b]Key Laboratory of Animal Antimicrobial Resistance Surveillance, Ministry of Agriculture and Rural Affairs, College of Veterinary Medicine, China Agricultural University, Beijing 100193, China
[c]College of Veterinary Medicine, Yangzhou University, Yangzhou, 225127, China

Running Head: heavy metals promoting spread of *tet*(X)-variant genes

#Address correspondence to Ran Wang, ranwang@jaas.ac.cn.

*Tao He and Jun Li contributed equally to this work. Author order was determined on the basis of seniority.

Word count of ABSTRACT: 223.
Word count of IMPORTANCE: 150.
Word count of main text (including Materials and Methods): 5451.

Supplementary Information (SI)

Table S1. Sampling information in this study.

Table S2. Previously reported *tet*(X)-variant-carrying plasmids.

Table S3. Quantitative polymerase chain reaction (qPCR) primers used in this study.

Table S4. The minimal inhibitory concentrations of heavy metals against the bacterial hosts.

Table S5. Detected concentrations of heavy metals among 96 organic fertilizer products.

Table S6. Detected concentrations of antimicrobials among 96 organic fertilizer products.

Table S7. Comparison of residual concentrations of pesticides in fertilizer samples of different origins.

Table S8. Relative abundances (copies/16S rRNA) of detected *tet*(X)-variants, two heavy metal resistance genes and plamid *rep* genes among 96 organic fertilizer products.

Table S9. Correlation by Spearman correlation analysis.

Table S10. Coexistence of *tet*(X3) or *tet*(X4) with the heavy metal resistance genes in the same plasmid obtained from NCBI.

Figure S1. The sampling sites and numbers of samples collected in this study.

Figure S2. Comparison of concentrations of antimicrobials in organic-fertilizer samples from different animals. SAs: sulfanilamides, FQs: fluoroquinolones, MAs: macrolides, TCs: tetracyclines, AMs: amphenicols.

Figure S3. Microbial diversity of *tet*(X)-variant-positive and *tet*(X)-variant-negative samples at Phylum level.

Figure S4. Comparison of genetic contexts of *tet*(X4)-bearing plasmids. A. Circular alignment of *tet*(X4)-bearing plasmid pRF14-1 with pRF14-1-like identified in this study. B. Circular alignment of *tet*(X4)-bearing plasmid pYPE10 with pYPE10-like-1 and pYPE10-like-2 identified in this study. pRF14-1-like plasmid and pYPE10-like-1/pYPE10-like-2 plasmid showed >99% coverage and >99% sequence identity with the previously reported conjugative *tet*(X4)-bearing plasmids pRF14-1 (GenBank accession no.: NZ_MT219822) and pYPE10 (GenBank accession no.: NZ_CP041449), respectively.

Figure S5. Redundancy analysis (RDA) of the relationships between *tet*(X)-variant

genes and concentrations of five heavy metals, five classes of antimicrobials, and all the pesticides. SAs: sulfanilamides, FQs: fluoroquinolones, MAs: macrolides, TCs: tetracyclines, AMs: amphenicols.

Figure S6. Heavy metals induced significant changes in the bacterial outer-membrane permeability. A. Changes in the outer membrane permeability of *tet*(X3)-positive *A. baumannii* ATCC17978 using the 1-N-Phenylnaphthylamine (NPN) probe on exposure to different heavy metals. B. Changes in the outer membrane permeability of *tet*(X4)-positive *E. coli* BL21 using the 1-N-Phenylnaphthylamine (NPN) probe on exposure to different heavy metals. Significant differences between outer membrane permeability with one concentration of the heavy metal and the corresponding control were tested using the unpaired *t*-test, *$p < 0.05$ **$p < 0.01$ ***$p < 0.001$.

Table S1. Sampling information in this study.

Provinces	Origin of raw materials for organic fertilizers				Number
	Chicken feces	Pig feces	Dairy feces	Plant waste[a]	Total
Zhejiang	3	5	0	8	16
Jiangsu	7	5	7	7	26
Hebei	4	3	11	0	18
Shandong	2	2	/	/	4
Henan	3	2	/	/	5
Liaoning	4	/	/	/	4
Heilongjiang	2	1	/	/	3
Hubei	4	4	/	/	8
Sichuan	5	5	/	/	10
Total	34	27	18	15	94

[a] Plant waste origin means the organic fertilizers were made from plant materials such as straw from wheat, corn and rice.

Table S2. Previously reported *tet*(X)-variant-carrying plasmids.

**Plasmid designation**	***tet*(X) variants**	**Bacterial host**	**Genbank accession no.**	**Incompatibility group or *rep* gene**	**Conjugative**	**T4SS type**
p34AB	*tet*(X3)	*Acinetobacter baumannii*	MK134375	*rep*-p34AB	Yes	Untypable
pB18-2	*tet*(X3)	*Acinetobacter indicus*	CP044457	*rep*-pB18-2	No	No
pCMG3-2-1	*tet*(X3)	*A. indicus*	CP044446	*rep*-pB18-2	No	No
pMMS9-2-1	*tet*(X3)	*A. indicus*	CP044451	*rep*-pB18-2	No	No
pHZE30-1-1	*tet*(X3)	*Acinetobacter schindleri*	CP044484	*rep*-pB18-2	No	No
pHZE33-1-1	*tet*(X3)	*A. schindleri*	CP044484	*rep*-pB18-2	No	No
pC15-1	*tet*(X3)	*A. indicus*	CP048655	*rep*-pB18-2	No	No
p18TQ-X3	*tet*(X3)	*A. indicus*	CP045132	*rep*-pB18-2	No	No
pAB17H194-1	*tet*(X3)	*Acinetobacter pittii*	CP040912	*rep*-pB18-2	No	No
pBDT2091-4	*tet*(X3)	*A. schindleri*	CP094838	*rep*-pB18-2	No	No
pYY76-1-2	*tet*(X4)	*Escherichia coli*	CP040929	IncX1	Yes	VirB/VirD (type P)
p54-tetX	*tet*(X4)	*Escherichia coli*	CP041286	IncX1	No	No
pYPE12-101k-tetX4	*tet*(X4)	*Escherichia coli*	CP041443	IncX1	No	No
pYPE10	*tet*(X4)	*E. coli*	CP041449	IncFIA(HI1)	Yes	Tra_F (type F)
pYPE3-92k-tetX4	*tet*(X4)	*E. coli*	CP041453	IncX1	No	No
pNT1F31-tetX4	*tet*(X4)	*E. coli*	CP045188	IncX1	No	No
p1916D18-1	*tet*(X4)	*E. coli*	CP045998	IncX1	Yes	VirB/VirD (type P)
p1916D6-2	*tet*(X4)	*E. coli*	CP046002	IncX1	Yes	VirB/VirD (type P)
p1919D3-1	*tet*(X4)	*E. coli*	CP046004	IncFIA(HI1)	Yes	Tra_F (type F)
p1919D62-1	*tet*(X4)	*E. coli*	CP046007	IncFIA(HI1)	Yes	Tra_F (type F)
pT16R-1	*tet*(X4)	*E. coli*	CP046717	IncFIA(HI1)	Yes	Tra_F (type F)
pZF31-tetX-119kb	*tet*(X4)	*E. coli*	CP047460	IncFIB(K)	No	No
pZF34-tetX-114kb	*tet*(X4)	*E. coli*	CP047466	IncFIB(K)	No	No
p2EC1-1	*tet*(X4)	*E. coli*	CP047572	IncI1	Yes	Tra_I (type I)
p94EC-2	*tet*(X4)	*E. coli*	CP047578	IncI1	Yes	Tra_I (type I)
pEC931_tetX	*tet*(X4)	*E. coli*	CP049121	IncX1	Yes	VirB/VirD (type P)
pT28R-1	*tet*(X4)	*E. coli*	CP049354	IncFIA(HI1)	Yes	Tra_F (type F)
pCD58-3-1	*tet*(X4)	*E. coli*	CP050037	IncX1	No	No
pCD63-2-1	*tet*(X4)	*E. coli*	CP050041	IncX1	No	No
pCD74-2-2	*tet*(X4)	*E. coli*	CP050046	IncX1	No	No
pSTB20-1T	*tet*(X4)	*E. coli*	CP050174	IncX1	No	No
pHNCF11W-tetX4	*tet*(X4)	*Escherichia fergusonii*	CP053047	IncX1	Yes	VirB/VirD (type P)
pSY3626C1_229k	*tet*(X4)	*E. coli*	CP058949	IncFIA(HI1)	Yes	Tra_F (type F)
pSY3626C1_315k	*tet*(X4)	*E. coli*	CP059044	IncFIA(HI1)	Yes	Tra_F (type F)
pSY3626_190k_tetX	*tet*(X4)	*E. coli*	CP059284	IncFIA(HI1)	Yes	Tra_F (type F)
pSal21GXH-tetX4	*tet*(X4)	*Salmonella enterica*	CP060586	IncFIA(HI1)	Yes	Tra_F (type F)
pb1-2L-1	*tet*(X4)	*Klebsiella pneumoniae*	CP072457	IncFIA(HI1)	Yes	Tra_F (type F)
pK-1L-1	*tet*(X4)	*K. pneumoniae*	CP072461	IncFIA(HI1)	Yes	Tra_F (type F)
p3Z-5L-2-X4	*tet*(X4)	*Klebsiella*	CP072517	IncFIA(HI1)	Yes	Tra_F

		quasipneumoniae				(type F)
pPK5074-tetX	*tet*(X4)	*E. coli*	CP072807	IncFII	Yes	Tra_F (type F)
pPK8217-tetX	*tet*(X4)	*E. coli*	CP080124	IncFII	Yes	Tra_F (type F)
pPK8568-tetX	*tet*(X4)	*E. coli*	CP080129	IncFII	Yes	Tra_F (type F)
pPK8277-tetX	*tet*(X4)	*E. coli*	CP080134	IncFII	Yes	Tra_F (type F)
pPK8241-tetX	*tet*(X4)	*E. coli*	CP080140	IncFII	Yes	Tra_F (type F)
pPK8276-tetX	*tet*(X4)	*E. coli*	CP080147	IncQ1	No	No
pPK8261-tetX	*tet*(X4)	*E. coli*	CP080156	IncFII	Yes	Tra_F (type F)
pPK8275-tetX	*tet*(X4)	*E. coli*	CP080164	IncFII	Yes	Tra_F (type F)
pPK8566-tetX	*tet*(X4)	*E. coli*	CP080175	IncFII	Yes	Tra_F (type F)
pPK5086-tetX	*tet*(X4)	*E. coli*	CP080371	IncFII	Yes	Tra_F (type F)
pLHC5-1-tetX	*tet*(X4)	*Citrobacter werkmanii*	CP084295	IncFIA(HI1)	Yes	Tra_F (type F)
pEC05-X4	*tet*(X4)	*E. coli*	MN436006	IncX1	No	No
pEC12-X4	*tet*(X4)	*E. coli*	MN436007	IncX1	No	No
pRB3-1_31K_tetX	*tet*(X4)	*E. coli*	MT197111	IncX1	No	No
pRF14-1	*tet*(X4)	*E. coli*	MT219822	IncX1	Yes	VirB/VirD (type P)
pRT18-1_294k_tetX	*tet*(X4)	*E. coli*	MT219824	IncFIA(HI1)	Yes	Tra_F (type F)
pXMPTW1-3	*tet*(X4)	*Citrobacter freundii*	MZ375739	IncX1	Yes	VirB/VirD (type P)
pSY3626_190k_tetX	*tet*(X4)	*E. coli*	NZ_JABXOE 010000002	IncFIA(HI1)	Yes	Tra_F (type F)
p47EC	*tet*(X4)	*E. coli*	NZ_MK1343 76	IncFIB(K)	Yes	Tra_F (type F)
pAB17H194-1	*tet*(X5)	*Acinetobacter pittii*	CP040912	*rep*-pAB17H194	No	No
pZN2-tetX-171kb	*tet*(X6)	*Proteus cibarius*	CP047350	IncA/C2	No	Tra_F (type F)
pZN3-tetX-171kb	*tet*(X6)	*Proteus vulgaris*	CP047345	IncA/C2	No	Tra_F (type F)
pZA25-tetX-168kb	*tet*(X6)	*Proteus mirabilis*	CP047353	IncA/C2	No	Tra_F (type F)

Table S3. qPCR primers used in this study.

Target genes	Genbank accession no. and location	Primers	Primer sequences (5′- 3′)	Length (bp)	References
16S rRNA	_	16S rRNA-F	CCTACGGGAGGCAGCAG	194	(1)
		16S rRNA-R	ATTACCGCGGCTGCTGG		
tet(X)/(X2)[a]	*tet*(X) (GU014535.1), *tet*(X2) (AJ311171)	*tet*(X)/(X2)-F	TGCGGCTAATGGCATCTCAC	227	(2)
		tet(X)/(X2)-R	GCTGCTACACATGACAACGTCGT		
tet(X3)	MK134375: 6174-7340	*tet*(X3)-F	GTGGATGCTTTGCTATTGTCTGA	125	(2)
		tet(X3)-R	TCTGTTGATTCGTCCTGCGTAT		
tet(X4)	MK134376: c1482-325	*tet*(X4)-F	TCGCTACAAAGAACTGATTCGTG	93	(2)
		tet(X4)-R	GGTCGCTTACTTCTCCAAGACTTAC		
tet(X5)	CP040912	*tet*(X5)-F	TGCCGTTGACCTACACAAAGG	161	(2)
		tet(X5)-R	TGTCAAAACGATTTTCGGGTC		
tet(X6)	PRJNA593823	*tet*(X6)-F	CCGGGCCTGTTGGATTAACA	117	(2)
		tet(X6)-R	GCAGATCAAGTGTCCCACCA		
IncX1 plasmid *rep* (*tet*(X4))	MT219822: c38652-37816	IncX1 *rep*-F	CATCGTTGCCTTTGTCTGCC	115	This study
		IncX1 *rep*-R	AATGCATATTCCCGAGCCGT		
IncFIA (HI1) plasmid *rep* (*tet*(X4))	CP041449: 45613-46368	*repE*-F	CGACTGGATCATGGAACGCT	111	This study
		repE-R	ATTGGTGTCCGGCTGTTGAT		
IncFII plasmid *rep* (*tet*(X4))	CP072807: 90246-91103	*repA*-F	ACATCACGTTCACACTGGCT	256	This study
		repA-R	TCCTGACGTTCTCTGTTCGC		
IncI1 plasmid *rep* (*tet*(X4))	CP047572: 46-1077	*repI1*-F	ACTTCACGAACAGCAGCAGA	84	This study
		repI1-R	TCATCCTCACGCAGAACACC		
IncFIB(K) plasmid *rep* (*tet*(X4))	MK134376: c16538-15528	*repA2*-F	CGGTCTCTTCGTCCCAACTC	187	This study
		RepA2-R	GCGAATTATCCCCAGCCAGA		
pB18-2 plasmid *rep* (*tet*(X3))	CP044457: c42212-41040	*rep*-pB18-2-F	TGAGGAGCCTGCTGATAATGG	160	This study
		rep-pB18-2-R	ATTACCCCCAGCCAGTTGAG		
p34AB plasmid *rep* (*tet*(X3))	MK134375: 134004-134528	*rep*-34AB-F	ACTCCCTCAATCAGCTCCCA	119	This study
		rep-34AB-R	GCTCAACGTGTTCTGGGACA		
pAB17H194-1 plasmid *rep* (*tet*(X5))	CP040912: c73828-72656	*rep*-pAB-F	CCGTTAAGAAAGGCCGTCCA	253	This study
		rep-pAB-R	TGAGAGTCAGTGAGGCATCC		
pZN2 plasmid *rep* (*tet*(X6))	CP047350: 37-1101	*rep*-pZN2-F	GGGACAGCGAGAGGAAATCC	152	This study
		rep-pZN2-R	CGTTCAGGTTGTACCCGTGA		
czcA	CP045132	*czcA*-F	ATTTATTGGGCCCGTCAGCA	211	This study
		czcA-R	GACGCACAATCCAGTCCTGA		
merT	MT219816	*merT*-F	GCTTGGATCGGCAACTTGAC	220	This study
		merT-R	TGACGTAGGGAAATCCGAGC		
dotB	MK134375: c256266-254971	*dotB*-F	TTTCTGGCATTCCCCCTCAC	108	This study
		dotB-R	CAGAACCAGTGATTCCGCCA		
dotC	MK134375: c257103-256294	*dotC*-F	ATGGGTTTAGTTGAGGGCCG	263	This study
		dotC-R	GGTGCAATTTGTTCAGGTTGGA		
dotI	MK134375: 260194-260877	*dotI*-F	TGGCTCAGAAAACAGGTGGA	101	This study
		dotI-R	GCAAGAACAGCACTCACGAT		
dotG	MK134375: 261860-263371	*dotG*-F	TGGTACGGAAATGGCGAACA	113	This study
		dotG-R	TCGGTGTTGTTACGTCAGCA		
traA_F (InFIB(K))	MK134376: 63626-63988	*traA*-F	ATATGCTTCGCCTGGTACGC	191	This study
		traA-R	CATGACAGCACCGACCAGAA		
traK_F (InFIB(K))	MK134376: 64903-65607	*traK*-F	TGCCACCGTCAACAAAAAGC	188	This study
		traK-R	TGGTCACAAGCAGGGATTCG		
traB_F (InFIB(K))	MK134376: 65607-67034	*traB*-F	TAACGGCAAAATCCTCGGCT	133	This study
		traB-R	TTTCAGGACATCACCAGCCC		
traC_F	MK134376:	*traC*-F	AATGAGCTTCCCGCAGTTCA	130	This study

(InFIB(K))	68946-71573	*traC*-R	GGACTCATTCGCCCCATTGA		
traG_F (InFIB(K))	MK134376: 79640-82462	*traG*-F	GGGATGTATGGCGATGCTCA	101	This study
		traG-R	TCAATACTTGCCCGACCACC		
virB2 (IncX1)	MT219822: 46274-46564	*virB2*-F	ACCCTGTCTGTCTTCATGGC	147	This study
		virB2-R	CGCAATGATAGTGATAGCTGGC		
virB4 (IncX1)	MT219822: 46588-49347	*virB3*-F	AGCTGCCTACTACCTTCCCA	133	This study
		virB3-R	TTTGACTCCACGGTTAGCCC		
virB8 (IncX1)	MT219822: 1087-1809	*virB8*-F	CGGAGGAATGTCAGGGATGG	129	This study
		virB8-R	GTCAACCTTTCAGCAACACCA		
virB9 (IncX1)	MT219822: 1815-2744	*virB9*-F	ACCCCCAAAATACGACCGTT	112	This study
		virB9-R	GACCAACCTTGCGGAAAACC		
virB10 (IncX1)	MT219822: 2741-3955	*virB10*-F	TTCGTGCAATGTCCAGCTCT	123	This study
		virB10-R	TGTCATCCCTCTGGCTCTGA		
virD4 (IncX1)	MT219822: 5015-6850	*virD4*-F	GGAACGGAAGGGGCAAAAAC	168	This study
		virD4-R	AGAGTTGCTTCGGGATGTGG		
traA_F (IncFIA(HI1))	CP041449: 176552-176905	*traA*-F	TCTCGGTGGCTTATTTCGGC	126	This study
		traA-R	GAGGAATACCAGCATCCAGGA		
traK_F (IncFIA(HI1))	CP041449: 178074-179306	*traK*-F	CTGGCACTACGACTGAGACC	119	This study
		traK-R	GTTATTTGACAACGCCCCGG		
traB_F (IncFIA(HI1))	CP041449: 179738-181096	*traB*-F	CCGACCCGAAACAGTCCAAT	135	This study
		traB-R	TTCACTTCCCGCAACAACCT		
traC_F (IncFIA(HI1))	CP041449: 183444-186125	*traC*-F	GCAATGGTGTGAAGGAGGGA	125	This study
		traC-R	CAGTCTGAAGGTGTGGGGTC		
traG_F (IncFIA(HI1))	CP041449: c108660-104671	*traG*-F	GTCACCACGAACCTTAGCCA	156	This study
		traG-R	GCTGTCACCACCCCATTGTA		

[a] *tet*(X) and *tet*(X2) could not be distinguished by qPCR as they shared >99% sequence identity with each other

Table S4. The minimal inhibitory concentrations (MICs) of heavy metals against bacterial hosts.

	MIC of heavy metals (μg/mL)					
	A. *baumannii* ATCC17978 -p34AB (*tet*(X3)$^+$)	A. *baumannii* 5AB	*E. coli* BL21-p47EC (*tet*(X4)$^+$)	*E. coli* BL21-pRF14-1 (*tet*(X4)$^+$)	*E. coli* BL21-pYPE10 (*tet*(X4)$^+$)	*E. coli* J53
As^{5+}	250	125	250	250	250	125
Pb^{2+}	15	15	15	15	15	15
Cd^{2+}	15	7.5	60	60	60	125
Cr^{6+}	15	3	30	15	30	15
Hg^{2+}	1.5	3	7.5	7.5	7.5	1.5

Table S5. Detected concentrations (mg/kg) of heavy metals among 96 organic fertilizer products.

	Hg	As	Cd	Cr	Pb	Total
Chinese National standard limits	≤2 mg/kg	≤15 mg/kg	≤3 mg/kg	≤150 mg/kg	≤50 mg/kg	
YJF-2020001	0.04	4.95	0.71	**199.46**[a]	14.43	219.59
YJF-2020002	0.10	0.41	0.32	2.55	0.92	4.30
YJF-2020003	ND[b]	4.03	1.13	23.14	18.07	46.37
YJF-2020004	0.19	5.26	1.15	50.57	6.44	63.62
YJF-2020005	0.30	3.86	0.64	15.17	2.82	22.80
YJF-2020006	ND	4.42	0.98	95.73	**66.00**	167.13
YJF-2020007	0.07	5.00	0.98	33.73	38.56	78.33
YJF-2020008	0.07	4.64	1.31	20.11	39.93	66.06
YJF-2020009	0.39	7.94	1.46	31.21	**92.14**	133.13
YJF-2020010	0.61	3.79	0.40	9.15	2.03	15.97
YJF-2020011	0.12	6.47	0.38	10.51	1.17	18.64
YJF-2020012	ND	8.96	0.33	6.63	0.46	16.37
YJF-2020013	0.09	1.60	0.63	37.93	13.17	53.42
YJF-2020014	0.02	**121.56**	0.09	10.52	3.48	135.67
YJF-2020015	0.06	5.37	**4.93**	15.42	12.48	38.26
YJF-2020016	0.13	**19.94**	1.22	59.16	**505.93**	586.39
YJF-2020017	0.03	6.21	0.37	29.73	23.85	60.18
YJF-2020018	0.19	4.20	0.22	38.71	9.29	52.60
YJF-2020019	0.02	2.02	0.68	12.48	16.17	31.36
YJF-2020020	0.05	10.83	0.09	10.87	3.48	25.32
YJF-2020021	0.04	5.12	1.14	21.26	45.02	72.58
YJF-2020022	0.01	1.40	0.05	4.50	7.33	13.28
YJF-2020023	0.15	5.77	0.45	75.80	24.59	106.75
YJF-2020024	ND	1.17	0.01	5.63	0.93	7.74
YJF-2020025	0.02	12.25	0.89	27.57	24.89	65.62
YJF-2020026	0.03	5.47	1.02	20.99[a]	6.48	33.98
YJF-2020027	0.04	3.23	0.14	25.85	7.57	36.83
YJF-2020028	0.02	8.44	**4.92**	42.91	16.33	72.61
YJF-2020029	ND	4.73	0.05	7.75	0.71	13.24
YJF-2020030	0.42	**17.76**	0.63	40.37	9.01	68.19
YJF-2020031	ND	9.53	0.06	6.11	0.33	16.03
YJF-2020032	0.21	6.15	1.12	77.40	18.19	103.08
YJF-2020033	0.24	5.23	0.21	42.42	11.69	59.79
YJF-2020034	ND	2.36	0.01	4.61	1.15	8.13
YJF-2020035	0.02	6.34	0.59	25.46	10.54	42.95
YJF-2020036	0.13	**27.72**	**11.42**	52.04	**251.19**	342.50
YJF-2020037	ND	5.62	0.10	8.43	2.87	17.01

YJF-2020038	0.26	9.67	0.20	26.65	4.05	40.82
YJF-2020039	0.09	7.16	2.71	48.99	42.43	101.38
YJF-2020040	0.09	4.52	0.27	22.24	15.72	42.83
YJF-2020041	0.49	5.59	0.04	3.62	1.00	10.74
YJF-2020042	0.04	8.68	1.43	28.88	45.55	84.58
YJF-2020043	0.04	6.03	1.13	25.94	46.73	79.88
YJF-2020044	0.02	6.03	0.65	40.46	26.74	73.89
YJF-2020045	ND	3.80	0.12	16.37	7.06	27.34
YJF-2020046	0.08	6.89	1.13	23.16	**62.11**	93.38
YJF-2020047	ND	2.99	0.04	7.30	15.75	26.08
YJF-2020048	ND	3.77	0.30	22.28	9.60	35.95
YJF-2020049	ND	4.18	0.12	13.37	7.27	24.93
YJF-2020050	0.32	2.93	0.34	21.51	28.12	53.22
YJF-2020051	0.29	6.46	1.05	60.06	7.89	75.77
YJF-2020052	0.05	11.23	0.59	28.00	23.50	63.36
YJF-2020053	0.06	5.95	0.63	23.44	42.89	72.97
YJF-2020054	0.17	4.53	0.61	21.81	26.20	53.33
YJF-2020055	0.01	4.06	0.31	31.76	11.43	47.58
YJF-2020056	ND	0.25	0.04	5.42	0.64	6.35
YJF-2020057	0.02	3.79	0.19	20.89	11.94	36.84
YJF-2020058	0.03	3.25	0.63	35.68	21.07	60.66
YJF-2020059	0.07	5.25	0.62	51.29	13.00	70.23
YJF-2020060	0.17	4.00	0.31	20.02	16.33	40.82
YJF-2020061	0.53	3.80	0.50	33.18	13.12	51.14
YJF-2020062	0.27	4.74	0.24	18.14	24.77	48.16
YJF-2020063	0.04	3.60	0.61	25.24	16.75	46.24
YJF-2020064	0.08	6.71	0.48	34.00	14.91	56.19
YJF-2020065	0.21	6.60	1.01	23.36	22.72	53.90
YJF-2020066	0.13	3.74	0.73	27.90	16.24	48.75
YJF-2020067	0.17	5.69	0.92	32.25	15.17	54.21
YJF-2020068	0.07	3.14	0.38	21.44	12.25	37.28
YJF-2020069	ND	3.90	0.62	40.61	13.67	58.80
YJF-2020070	ND	0.04	0.08	2.19	0.23	2.53
YJF-2020071	0.57	7.58	1.63	**250.00**	22.58	282.36
YJF-2020072	0.04	2.88	0.33	35.31	14.96	53.52
YJF-2020073	0.07	4.70	0.37	24.54	12.30	41.99
YJF-2020074	0.04	4.52	0.35	23.25	11.95	40.11
YJF-2020075	0.01	0.22	0.50	7.34	4.20	12.26
YJF-2020076	ND	0.96	0.01	2.25	1.42	4.64
YJF-2020077	ND	0.03	0.03	3.05	0.83	3.94
YJF-2020078	ND	3.74	0.25	15.00	8.82	27.80

YJF-2020079	1.47	6.35	0.85	63.66	28.94	101.27
YJF-2020080	0.11	11.41	2.80	27.39	49.35	91.06
YJF-2020081	ND	0.13	0.11	9.52	0.36	10.12
YJF-2020082	ND	0.87	0.20	13.25	5.31	19.62
YJF-2020083	0.01	4.55	0.35	20.58	7.60	33.09
YJF-2020084	0.14	5.72	1.31	27.24	26.80	61.21
YJF-2020085	ND	1.68	0.63	43.31	12.16	57.78
YJF-2020086	0.75	9.44	1.84	37.17	19.85	69.05
YJF-2020087	0.27	8.52	0.51	42.90	15.65	67.86
YJF-2020088	0.07	14.89	1.84	51.26	27.21	95.26
YJF-2020089	0.02	7.92	1.83	27.43	43.71	80.91
YJF-2020090	0.77	4.10	0.91	13.93	13.30	33.02
YJF-2020091	0.14	8.93	0.83	14.89	12.75	37.54
YJF-2020092	0.36	10.00	2.40	27.53	28.09	68.38
YJF-2020093	**2.07**	**16.16**	1.49	37.44	21.45	78.60
YJF-2020094	0.29	8.39	2.74	30.41	**83.48**	125.31

[a] The values in bold were that which exceeded the limits requested in the national standards.

[b] ND means not detected using the methods in this study.

Table S6. Detected concentrations of antimicrobials among 96 organic fertilizer products.

	**SD**[a]	**SMZ**	**SM2**	**SMM**	**SQ**	**ENR**	**CIP**	**DFL**	**SAR**	**AZM**	**TIL**	**TYL**	**TET**	**OTC**	**CTET**	**DOX**	**THI**	**FFC**
YJF-2020001[b]										8.52								
YJF-2020002																		
YJF-2020003																		
YJF-2020004										8.74								
YJF-2020005										1.93								
YJF-2020006																		
YJF-2020007																		
YJF-2020008							4.99											
YJF-2020009											7.36			**137.87**[c]	**121.44**			
YJF-2020010																		
YJF-2020011																		
YJF-2020012																		
YJF-2020013																		
YJF-2020014					5.76													
YJF-2020015																		
YJF-2020016																59.32		
YJF-2020017														13.78	8.51			
YJF-2020018																		
YJF-2020019																		
YJF-2020020																		

YJF-2020021							27.64											
YJF-2020022											2.07							
YJF-2020023										8.38								
YJF-2020024																		
YJF-2020025																		
YJF-2020026																		
YJF-2020027					3.18													
YJF-2020028					11.07		13.86	**480.7**										
YJF-2020029																		
YJF-2020030																		
YJF-2020031																		
YJF-2020032																		
YJF-2020033					7.9					8.93								
YJF-2020034																		
YJF-2020035																		
YJF-2020036							**118.46**											
YJF-2020037										8.5								
YJF-2020038																		
YJF-2020039								**102.09**		8.51								
YJF-2020040											7.72							
YJF-2020041																		
YJF-2020042																		

YJF-2020043								**212.48**		8.73								
YJF-2020044	5.03																	
YJF-2020045																		
YJF-2020046																		
YJF-2020047																		
YJF-2020048																		
YJF-2020049											7.4							
YJF-2020050																		
YJF-2020051																		
YJF-2020052										8.51								
YJF-2020053																		
YJF-2020054																		
YJF-2020055										8.36								
YJF-2020056																		
YJF-2020057																		
YJF-2020058																		
YJF-2020059																		
YJF-2020060																		
YJF-2020061										8.49								
YJF-2020062																		
YJF-2020063							11.47											
YJF-2020064							11.93							44.2				

YJF-2020065																		
YJF-2020066																		
YJF-2020067																		
YJF-2020068																		
YJF-2020069																		
YJF-2020070																		
YJF-2020071										8.53								
YJF-2020072								53.7										
YJF-2020073														15.01				
YJF-2020074														15.75	15.68			
YJF-2020075																		
YJF-2020076																		
YJF-2020077							29.54											
YJF-2020078																		
YJF-2020079														16.96				
YJF-2020080																		
YJF-2020081																		
YJF-2020082																		
YJF-2020083																		
YJF-2020084										8.85								
YJF-2020085																		
YJF-2020086																		

YJF-2020087																		
YJF-2020088																64.16		
YJF-2020089																		
YJF-2020090																	44.46	
YJF-2020091																		
YJF-2020092					6.33													
YJF-2020093														23.1	56.31			
YJF-2020094								**114.36**										

[a] SD, sulfadiazine; SMZ, sulfamethoxazole; SM2, sulfamethazine; SMM, sulfamonomethoxine; SQ, sulfaquinoxaline; ENR, enrofloxacin; CIP, ciprofloxacin; DFL, dafloxacin; SAR, sarafloxacin; AZM, azithromycin; TIL, tilmicosin; TYL, tylosin; TET, tetracycline; OTC, oxytetracycline; CTET, chlortetracycline; DOX, doxycycline; THI, thiamphenicol; FFC, florfenicol.

[b] Blank means not detected using the detection methods in this study.

[c] The values in bold were the concentrations that were more than 100 μg/kg.

Table S7. Comparison of residual concentrations of pesticides in fertilizer samples of different origins.

	Chicken feces origin (n=34)			Pig feces origin (n=27)			Dairy feces origin (n=18)			Plant waste origin (n=15)			Total (n=94)		
	Detection rate (%)	Concentration ranges (μg/kg)	Mean[a] (μg/kg)	Detection rate (%)	Concentration ranges (μg/kg)	Mean (μg/kg)	Detection rate (%)	Concentration ranges (μg/kg)	Mean (μg/kg)	Detection rate (%)	Concentration ranges (μg/kg)	Mean (μg/kg)	Detection rate (%)	Concentration ranges (μg/kg)	Mean (μg/kg)
avermectin	0	−	−	3.70	3.81	3.81	5.56	13.69	13.69	0	−	−	2.13	3.81-13.69	8.75
chlorpyrifos	2.94	8.41	8.41	0	−	−	0	−	−	6.67	20.66	20.66	2.13	8.41-20.66	14.54
fenpropathrin	2.94	5.66	5.66	0	−	−	0	−	−	6.67	12.63	12.63	1.06	2.84-2.84	2.84
imidacloprid	2.94	2.16	2.16	0	−	−	0	−	−	13.33	2.21-19.64	10.93	3.19	2.16-19.64	8
tetramethrin	0	−	−	0	−	−	0	−	−	6.67	2.48	2.48	1.06	2.48	2.48
triazophos	0	−	−	0	−	−	0	−	−	40	2.32-13.88	6.26	6.38	2.32-13.88	6.26
tebuconazole	0	−	−	3.70	5.02	5.02	5.56	2.62	2.62	20	2.22-86.68	30.70	5.32	2.22-86.68	19.95
3-OH-Carbofuran	0	−	−	0	−	−	0	−	−	6.67	5.31	5.31	1.06	5.31	5.31
bifenthrin	0	−	−	0	−	−	0	−	−	6.67	2.84	2.84	1.06	2.84	2.84
carbendazim	0	−	−	0	−	−	0	−	−	20	2.28-2.93	2.67	3.19	2.28-2.93	2.67
dichlorvos	2.94	13.29	13.29	7.41	8.51	8.53	11.11	3.67	3.70	6.67	4.99	4.99	6.38	3.67-13.29	7.12
diethofencarb	0	−	−	3.70	2.55	2.55	0	−	−	26.67	2.44-13.74	6.34	5.32	2.44-13.74	5.58
isocarbophos	2.94	2.40	2.40	0	−	−	0	−	−	26.67	2.24-8.10	3.79	5.32	2.24-8.10	3.51
prochloraz	0	−	−	0	−	−	0	−	−	20	2.65-4.36	3.35	3.19	2.65-4.36	3.35
triazophos	0	−	−	0	−	−	0	−	−	6.67	3.29	3.29	1.06	3.29	3.29
cyromazine	32.35	4.62− 828.76	91.13	33.33	3.27-653.69	105.01	38.89	3.60-148.31	39.17	0	−	−	28.72	3.27-828.76	82.29
methyl parathion	0	−	−	0	−	−	0	−	−	13.33	10.78-10.99	10.89	2.13	10.78-10.99	10.89
carbaryl	0	−	−	0	−	−	0	−	−	6.67	2.71	2.71	1.06	2.71	2.71

[a] The mean indicated the average value of the detected concentrations of pesticides.

Table S8. Relative abundances (copies/16S rRNA) of detected *tet*(X)-variants, two heavy metal resistance genes and plasmid *rep* genes among 96 organic fertilizer samples.

		tet(X)-variant genes					Heavy metal resistance genes		*rep* genes from *tet*(X)-variant-carrying plasmids								
									tet(X4)					*tet*(X3)		*tet*(X5)	*tet*(X6)
	Origins	*tet*(X)/(X2)	*tet*(X3)	*tet*(X4)	*tet*(X5)	*tet*(X6)	*czcA*	*merT*	IncX1	IncFIA(HI1)	IncFII	IncI1	IncFIB(K)	pB18-2	p34AB	pAB17H194-1	pZN2
YJF-2020001	Chicken feces		5.10×10^{-4}				4.28×10^{-4}							6.56×10^{-4}			
YJF-2020002	plant waste	8.64×10^{-5}															
YJF-2020003	plant waste																
YJF-2020004	Pig feces				5.21×10^{-4}									4.58×10^{-4}			
YJF-2020005	plant waste							4.36×10^{-4}									
YJF-2020006	Pig feces						6.27×10^{-3}		2.86×10^{-4}	4.73×10^{-4}							
YJF-2020007	Pig feces	2.81×10^{-4}															
YJF-2020008	plant waste																
YJF-2020009	Chicken feces	1.44×10^{-4}	9.40×10^{-5}					7.55×10^{-4}						2.13×10^{-3}			
YJF-2020010	plant waste							6.75×10^{-4}									
YJF-2020011	plant waste																
YJF-2020012	plant waste	7.36×10^{-5}															
YJF-2020013	plant waste																
YJF-2020014[a] pYPE10-like-1	Pig feces		5.42×10^{-4}	2.78×10^{-4}			6.59×10^{-4}			7.56×10^{-4}			6.32×10^{-4}				
YJF-2020015	Chicken feces		2.30×10^{-4}												3.67×10^{-4}		
YJF-2020016 pYPE10-like-2	Pig feces			4.74×10^{-4}		3.10×10^{-4}			8.56×10^{-4}	8.27×10^{-4}			7.41×10^{-4}				

YJF-2020017	Dairy feces						8.17×10^{-4}										
YJF-2020018	Dairy feces								3.77×10^{-4}								
YJF-2020019	plant waste																
YJF-2020020	Pig feces	1.73×10^{-4}					4.73×10^{-3}										
YJF-2020021	Dairy feces	3.88×10^{-4}															
YJF-2020022	plant waste																
YJF-2020023	Dairy feces								2.93×10^{-4}	6.89×10^{-4}							
YJF-2020024	plant waste	3.57×10^{-4}															
YJF-2020025	Pig feces						7.50×10^{-3}		4.24×10^{-3}								
YJF-2020026	plant waste																
YJF-2020027	plant waste																
YJF-2020028	Chicken feces		3.76×10^{-4}				2.33×10^{-2}							6.42×10^{-4}			
YJF-2020029	Dairy feces																
YJF-2020030	Pig feces			3.29×10^{-4}					6.93×10^{-4}	6.18×10^{-4}			5.33×10^{-4}				
YJF-2020031	Pig feces						7.52×10^{-4}		6.62×10^{-4}								
YJF-2020032	Chicken feces	1.43×10^{-4}						2.17×10^{-4}									
YJF-2020033	Chicken feces																
YJF-2020034	plant waste	2.62×10^{-4}															
YJF-2020035	Dairy feces						4.94×10^{-3}										
YJF-2020036	Chicken feces			5.30×10^{-4}					8.25×10^{-4}								
YJF-2020037	Dairy feces																
YJF-2020038	Pig feces														6.39×10^{-4}		2.47×10^{-4}
YJF-2020039	Chicken feces	2.90×10^{-4}															
YJF-2020040	Chicken feces						3.28×10^{-3}		6.13×10^{-4}								

YJF-2020041	plant waste																
YJF-2020042	Chicken feces		3.73×10^{-4}				2.47×10^{-3}							4.66×10^{-4}			
YJF-2020043	Dairy feces		2.12×10^{-4}														
YJF-2020044	Dairy feces												8.27×10^{-4}				
YJF-2020045	Dairy feces										8.54×10^{-4}						
YJF-2020046	Pig feces				8.94×10^{-5}											2.56×10^{-4}	
YJF-2020047	Dairy feces									7.31×10^{-4}				5.44×10^{-4}			
YJF-2020048	Dairy feces													8.27×10^{-4}			
YJF-2020049	Dairy feces										6.44×10^{-4}						
YJF-2020050	Chicken feces	3.47×10^{-4}															
YJF-2020051	Chicken feces								8.35×10^{-4}								
YJF-2020052	Chicken feces																
YJF-2020053	Pig feces		2.95×10^{-4}				1.93×10^{-3}										
YJF-2020054	Pig feces												8.94×10^{-4}				
YJF-2020055	Chicken feces								3.29×10^{-4}								
YJF-2020056	Dairy feces	3.78×10^{-4}															
YJF-2020057	Dairy feces																
YJF-2020058	Dairy feces													3.16×10^{-4}			
YJF-2020059	Dairy feces													5.73×10^{-4}			
YJF-2020060	Dairy feces																
YJF-2020061	Chicken feces						5.22×10^{-3}										
YJF-2020062	Chicken feces								7.52×10^{-4}								
YJF-2020063	Pig feces									6.23×10^{-4}							
YJF-2020064	Pig feces									5.79×10^{-4}							

YJF-2020065	Chicken feces																
YJF-2020066	Chicken feces								6.53×10^{-4}								
YJF-2020067	Pig feces																
YJF-2020068	Pig feces						6.17×10^{-3}										
YJF-2020069	Pig feces																
YJF-2020070	Chicken feces												8.93×10^{-4}				
YJF-2020071	Chicken feces		6.77×10^{-4}				5.10×10^{-4}							9.53×10^{-4}			
YJF-2020072	Pig feces																
YJF-2020073	Chicken feces													6.62×10^{-4}			
YJF-2020074	Chicken feces												7.16×10^{-4}				
YJF-2020075	Chicken feces	3.72×10^{-4}															
YJF-2020076	Chicken feces															3.32×10^{-4}	
YJF-2020077	Chicken feces																
YJF-2020078	Chicken feces		4.66×10^{-4}				8.73×10^{-3}								5.76×10^{-4}		
**YJF-2020079 pRF14-1-like**	Pig feces	3.82×10^{-4}		6.23×10^{-4}				2.60×10^{-3}	8.46×10^{-4}								
YJF-2020080	Chicken feces						8.91×10^{-3}										
YJF-2020081	Chicken feces																
YJF-2020082	Chicken feces	3.51×10^{-4}															
YJF-2020083	Pig feces																
YJF-2020084	Pig feces						2.71×10^{-3}										
YJF-2020085	Chicken feces	3.65×10^{-4}															
YJF-2020086	Chicken feces							6.27×10^{-4}									
YJF-2020087	Chicken feces	3.24×10^{-4}															

YJF-2020088	Pig feces																
YJF-2020089	Pig feces		3.55×10^{-4}				4.27×10^{-3}										
YJF-2020090	Pig feces							5.89×10^{-4}									
YJF-2020091	Chicken feces																
YJF-2020092	Chicken feces													1.48×10^{-3}			
YJF-2020093	Pig feces		7.90×10^{-5}	6.84×10^{-4}				4.63×10^{-3}	9.28×10^{-4}								
YJF-2020094	Pig feces		4.63×10^{-4}				9.80×10^{-3}										

[a] The designations in bold mean that the *tet*(X4)-carrying plasmid was captured by *E. coli* from these samples.

Table S9. Correlation by Spearman correlation analysis.

	∑*tet*(X)-variants[a]	*tet*(X)/(X2)	*tet*(X3)	*tet*(X4)	*tet*(X5)	*tet*(X6)
czcA	0.178	-0.179	0.474***	-0.0506	-0.0759	-0.0534
merT	0.107	0.147	0.0828	0.266**	-0.0449	-0.0316
IncX1	0.100	-0.063	-0.091	0.318**	-0.034	0.152
IncFIA(HI1)	0.182	-0.130	0.0599	0.293**	-0.0361	0.427
IncFIB(K)	0.127	-0.121	0.0316	0.231*	-0.0335	0.357
pB18-2	0.113	-0.0513	0.264*	-0.0800	0.107	-0.0333
p34AB	0.0644	-0.0766	0.221*	-0.0442	-0.0213	-0.0184
Hg	0.0738	-0.0584	0.045	0.241*	0.0907	0.0635
As	0.232*	-0.148	0.285**	0.383***	0.0701	0.17
Cd	0.283**	-0.0786	0.325**	0.166	0.152	0.117
Cr	0.191	-0.0859	0.152	0.234*	0.0854	0.151
Pb	0.197	-0.0541	0.260*	0.158	0.0415	0.178
Total HMs[b]	0.369***[c]	-0.0597	0.389***	0.346***	0.135	0.178
DFL	0.201	-0.00119	0.321**	-0.0618	-0.0349	-0.0246
OTC	0.0801	0.0775	0.102	0.275**	-0.0418	-0.0294
DOX	0.0848	-0.0712	-0.0588	0.257*	-0.0217	0.696****

[a] ∑*tet*(X)-variants means the total amount of *tet*(X)/(X2), *tet*(X3), *tet*(X4), *tet*(X5) and *tet*(X6).

[b] HMs, heavy metals; DFL, dafloxacin; OTC, oxytetracycline; DOX, doxycycline;

[c] Values indicate the Spearman correlation coefficient (R). * means the significant level at the 0.05 level (p<0.05, two-tailed); ** means the significant level at the 0.01 level (p<0.01, 2-tailed); *** means the significant level at the 0.001 level (p<0.001, 2-tailed), **** means the significant level at the 0.0001 level (p<0.0001, 2-tailed). The chemical contaminants which has no value with significant difference are not shown.

Table S10. Coexistence of *tet*(X3) or *tet*(X4) with the heavy metal resistance genes in the same plasmid obtained from NCBI.

	tet(X)-variant gene	Bacterial species	Animal origin	Heavy metal resistance genes	Heavy metal resistance phenotype	Genbank no.
pCMG3-2-1	*tet*(X3)	*A. indicus*	goose	***czcC***, *B*, ***A***, *D*	Cd/Zn/Co	NZ_CP044446
pHZE33-1-1	*tet*(X3)	*A. schindleri*	goose	***czcC***, *B*, ***A***, *D*	Cd/Zn/Co	CP044475
pHZE23-1-1	*tet*(X3)	*A. schindleri*	goose	***czcC***, *B*, ***A***, *D*	Cd/Zn/Co	NZ_CP044464
pFS42-2-1	*tet*(X3)	*A. indicus*	duck	*cusA*/***czcA***	Cd/Zn/Co	CP046596
p10FS3-1-3	*tet*(X3)	*Acinetobacter* spp.	pig	***czcC***, *B*, ***A***, *D*	Cd/Zn/Co	CP039146
pYH12207-2	*tet*(X3)	*A. piscicola*	pig	*cusA*/***czcA***	Cd/Zn/Co	CP048661
p10FS3-1-3	*tet*(X3)	*Acinetobacter* spp.	pig	*copA*/*copB*， *cusA*/***czcA***	Cu、Cd/Zn/Co	CP039146
pMMS9-2-1	*tet*(X3)	*A. indicus*	soil	*cusA*/***czcA***	Cd/Zn/Co	CP044451
pAI01	*tet*(X3)	*A. indicus*	manure	*copA*/*copB*, ***czcC***, *B*, ***A***, *D*	Cu、Cd/Zn/Co	CP044019
pAT205	*tet*(X3)	*A. towneri*	pig	*copA*/*copB*, ***czcC***, *B*, ***A***, *D*	Cu、Cd/Zn/Co	CP048015
p18TQ-X3	*tet*(X3)	*A. indicus*	cow	***czcC***, *B*, ***A***, *D*, *merR*	Cd/Zn/Co、mercury	CP045132
pB18-2	*tet*(X3)	*A. indicus*	pigeon	*copA*/*copB*, ***czcC***, *B*, ***A***, *D*	Cu、Cd/Zn/Co	CP044457
pYUSHP10-1	*tet*(X3)	*Acinetobacter* spp.	pig	*copA*/*copB*, ***czcC***, *B*, ***A***, *D*	Cu、Cd/Zn/Co	MT107270
pNT1F10-tetX4	*tet*(X4)	*Escherichia coli*	Pig	*merD*/ *merC*/ *merP*/ ***merT***	Hg	NZ_CP075463
pNT1N34-tetX4	*tet*(X4)	*Escherichia coli*	Pig	*merD*/ *merC*/ *merP*/ ***merT***	Hg	NZ_CP075467

pNT1W22-tetX4	*tet*(X4)	*Escherichia coli*	Pig	***merT****/merD*	Hg	NZ_CP075470
pNT1F25-tetX4	*tet*(X4)	*Escherichia coli*	pig	*merD/merC/merP/****merT***	Hg	NZ_CP075471
pNT1N28-tetX4	*tet*(X4)	*Escherichia coli*	Pig	*merD/ merC/ merP/* ***merT***	Hg	NZ_CP075473
pNT1W25-tetX4	*tet*(X4)	*Escherichia coli*	Pig	*merD/merC/merP/****merT***	Hg	NZ_CP075479
pNT1N31-tetX4	*tet*(X4)	*Escherichia coli*	Pig	*merD/merC/merP/****merT***	Hg	NZ_CP075481
pNT1N25-tetX4	*tet*(X4)	*Escherichia coli*	Pig	*merD/merC/merP/****merT***	Hg	NZ_CP075485
pNT1F34-tetX4	*tet*(X4)	*Escherichia coli*	Pig	*merD/merC/merP/****merT***	Hg	NZ_CP075486
pT16R-2	*tet*(X4)	*Escherichia coli*	dog	*merE/merD/merC/merP/****merT****/mntB*	Hg/Mn	NZ_CP046718
pSTB20-1T	*tet*(X4)	*Escherichia coli*	pig	*merD/merC/merP/****merT***	Hg	NZ_CP050174
pCD63-2-1	*tet*(X4)	*Escherichia coli*	broiler chicken	***merT****/merP/merC/merD/merE*	Hg	NZ_CP050041

Figure S1.

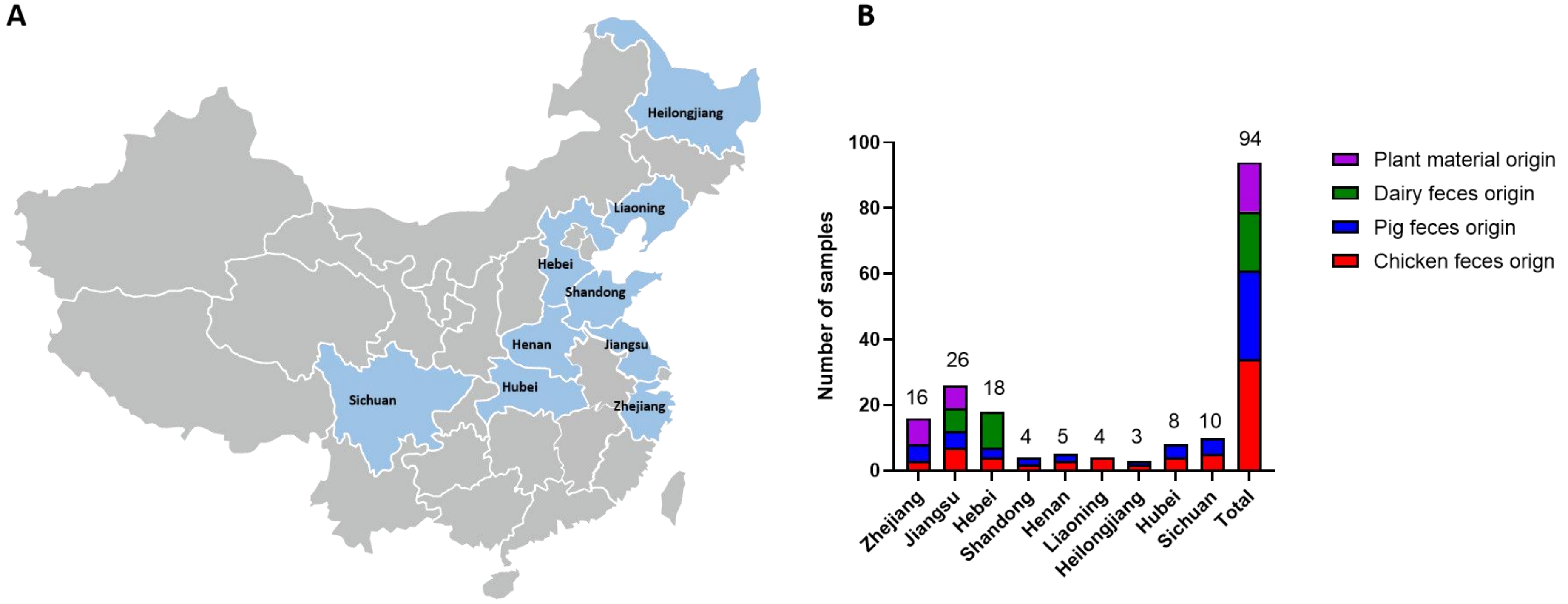
A
Heilongjiang
Liaoning
Hebei
Shandong
Henan
Jiangsu
Sichuan
Hubei
Zhejiang
B
Number of samples
100
80
60
40
20
0
16
26
18
4
5
4
3
8
10
94
Zhejiang
Jiangsu
Hebei
Shandong
Henan
Liaoning
Heilongjiang
Hubei
Sichuan
Total
Plant material origin
Dairy feces origin
Pig feces origin
Chicken feces orign

Figure S2.

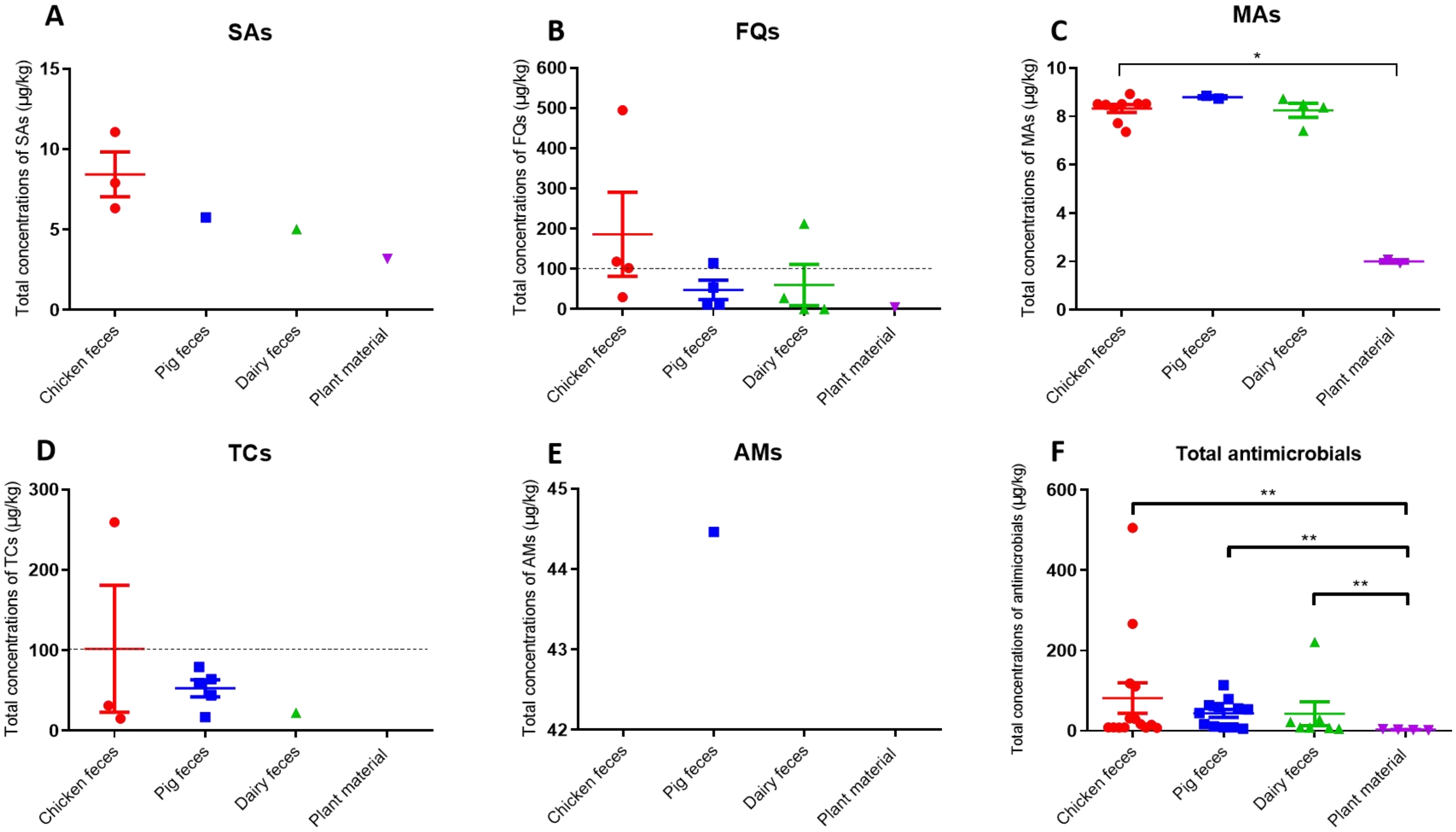

A
SAs
Total concentrations of SAs (µg/kg)
15
10
5
0
Chicken feces
Pig feces
Dairy feces
Plant material
B
FQs
Total concentrations of FQs (µg/kg)
600
500
400
300
200
100
0
Chicken feces
Pig feces
Dairy feces
Plant material
C
MAs
*
Total concentrations of MAs (µg/kg)
10
8
6
4
2
0
Chicken feces
Pig feces
Dairy feces
Plant material
D
TCs
Total concentrations of TCs (µg/kg)
300
200
100
0
Chicken feces
Pig feces
Dairy feces
Plant material
E
AMs
Total concentrations of AMs (µg/kg)
45
44
43
42
Chicken feces
Pig feces
Dairy feces
Plant material
F
Total antimicrobials
**
**
**
Total concentrations of antimicrobials (µg/kg)
600
400
200
0
Chicken feces
Pig feces
Dairy feces
Plant material

Figure S3.

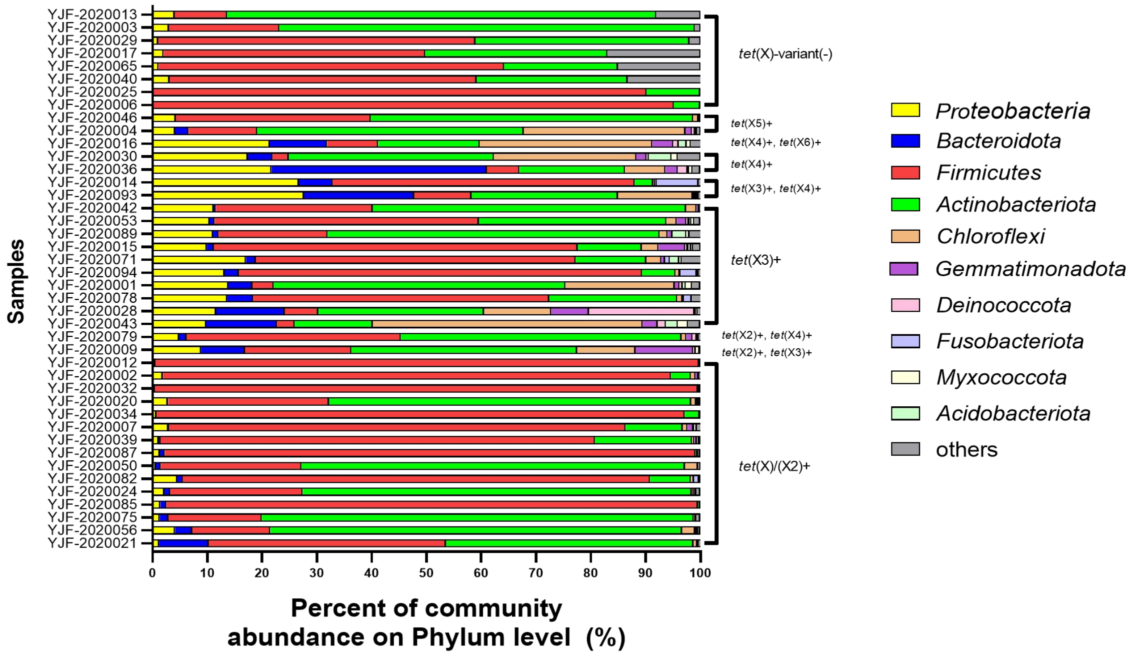

Samples
YJF-2020013
YJF-2020003
YJF-2020029
YJF-2020017
YJF-2020065
YJF-2020040
YJF-2020025
YJF-2020006
YJF-2020046
YJF-2020004
YJF-2020016
YJF-2020030
YJF-2020036
YJF-2020014
YJF-2020093
YJF-2020042
YJF-2020053
YJF-2020089
YJF-2020015
YJF-2020071
YJF-2020094
YJF-2020001
YJF-2020078
YJF-2020028
YJF-2020043
YJF-2020079
YJF-2020009
YJF-2020012
YJF-2020002
YJF-2020032
YJF-2020020
YJF-2020034
YJF-2020007
YJF-2020039
YJF-2020087
YJF-2020050
YJF-2020082
YJF-2020024
YJF-2020085
YJF-2020075
YJF-2020056
YJF-2020021
0 10 20 30 40 50 60 70 80 90 100
Percent of community abundance on Phylum level (%)
tet(X)-variant(-)
tet(X5)+
tet(X4)+, tet(X6)+
tet(X4)+
tet(X3)+, tet(X4)+
tet(X3)+
tet(X2)+, tet(X4)+
tet(X2)+, tet(X3)+
tet(X)/(X2)+
Proteobacteria
Bacteroidota
Firmicutes
Actinobacteriota
Chloroflexi
Gemmatimonadota
Deinococcota
Fusobacteriota
Myxococcota
Acidobacteriota
others

Figure S4.

A **B**

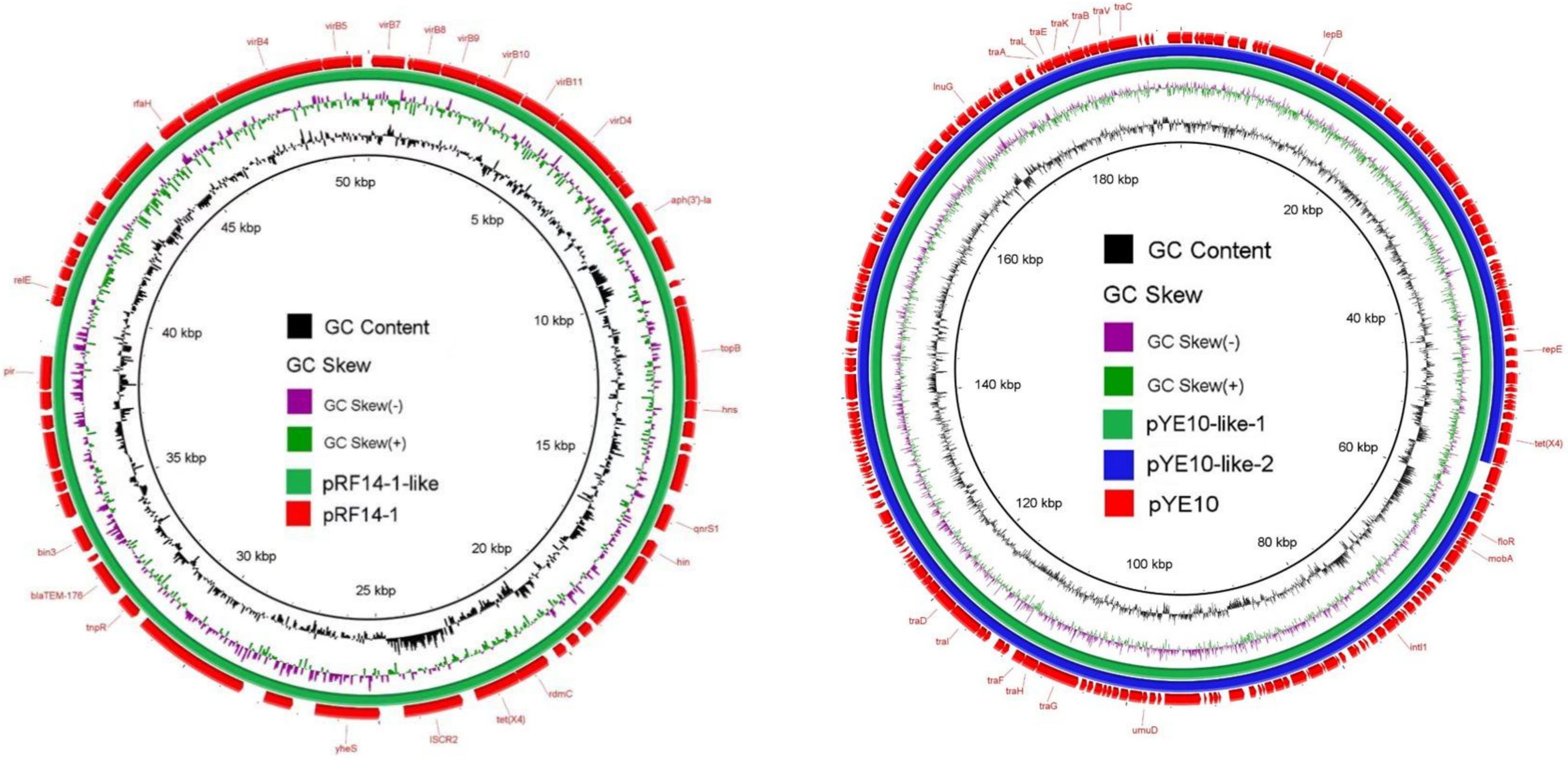
virB4
virB5
virB7
virB8
virB9
virB10
virB11
virD4
rfaH
aph(3')-Ia
relE
topB
pir
hns
qnrS1
hin
bin3
blaTEM-176
tnpR
rdmC
tet(X4)
ISCR2
yheS
50 kbp
5 kbp
10 kbp
15 kbp
20 kbp
25 kbp
30 kbp
35 kbp
40 kbp
45 kbp
GC Content
GC Skew
GC Skew(-)
GC Skew(+)
pRF14-1-like
pRF14-1
traA
traL
traE
traK
traB
traV
traC
lnuG
lepB
repE
tet(X4)
floR
mobA
intI1
umuD
traG
traH
traF
traI
traD
180 kbp
20 kbp
40 kbp
60 kbp
80 kbp
100 kbp
120 kbp
140 kbp
160 kbp
GC Content
GC Skew
GC Skew(-)
GC Skew(+)
pYE10-like-1
pYE10-like-2
pYE10

Figure S5.

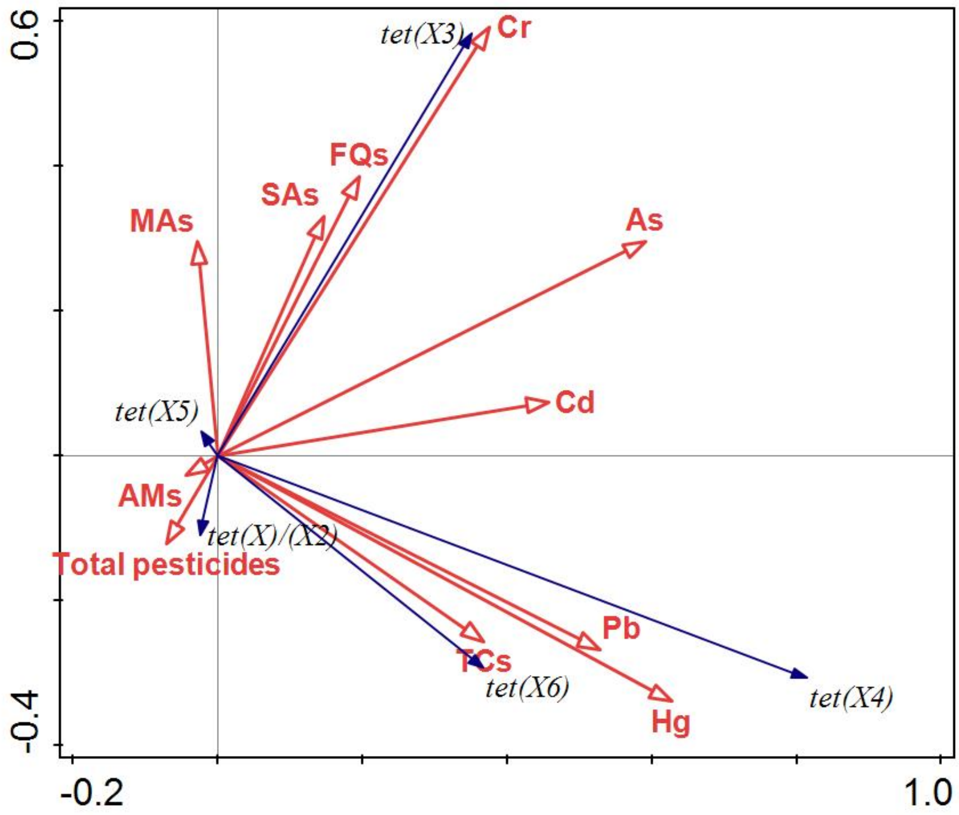

0.6
-0.4
-0.2
1.0
tet(X3)
Cr
FQs
SAs
MAs
As
Cd
tet(X5)
AMs
tet(X)/(X2)
Total pesticides
Pb
TCs
tet(X6)
tet(X4)
Hg

Figure S6.

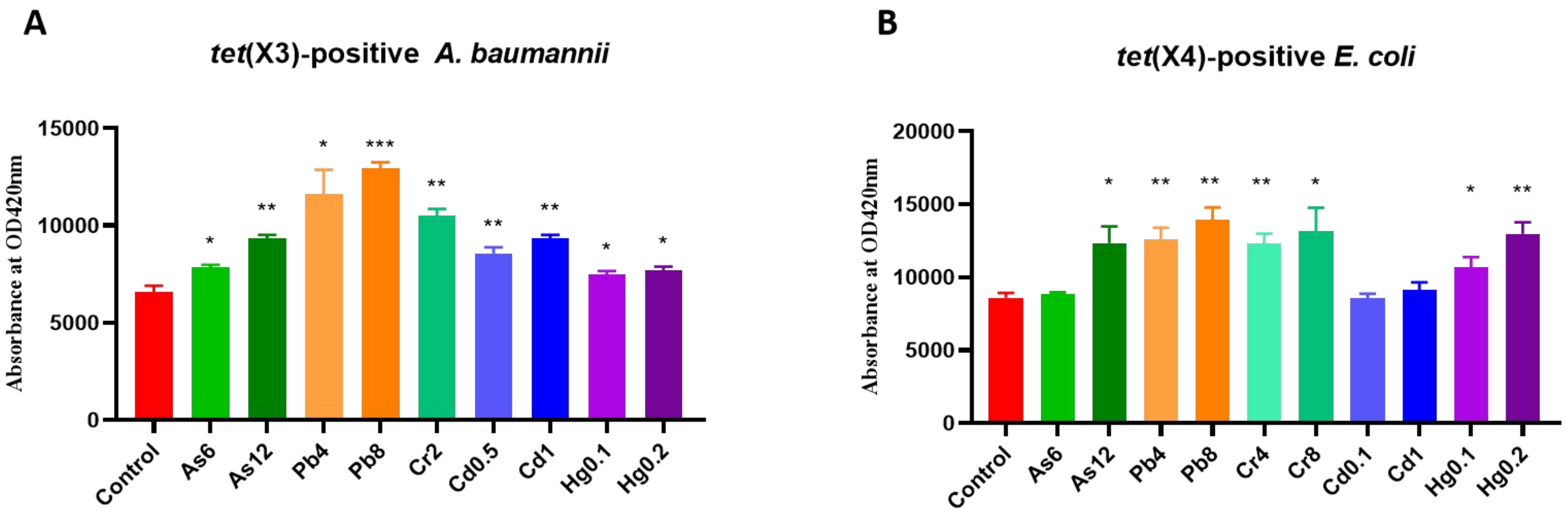
A
tet(X3)-positive *A. baumannii*
Absorbance at OD420nm
15000
10000
5000
0
*
**
*
***
**
**
**
*
*
Control
As6
As12
Pb4
Pb8
Cr2
Cd0.5
Cd1
Hg0.1
Hg0.2
B
tet(X4)-positive *E. coli*
Absorbance at OD420nm
20000
15000
10000
5000
0
*
**
**
**
*
*
**
Control
As6
As12
Pb4
Pb8
Cr4
Cr8
Cd0.1
Cd1
Hg0.1
Hg0.2

REFERENCES

1. Qiu, Z., Yu, Y., Chen, Z., Jin, M., Yang, D., Zhao, Z., Wang, J., Shen, Z., Wang, X., Qian, D., Huang, A., Zhang, B., Li, J.W., 2012. Nanoalumina promotes the horizontal transfer of multiresistance genes mediated by plasmids across genera. Proc Natl Acad Sci U S A. 109(13), 4944-4949. https://doi.org/10.1073/pnas.1107254109.

2. He, T., Wei, R.C., Zhang, L., Gong, L., Zhu, L., Gu, J., Fu, Y.L., Wang, Y., Liu, D.J., Wang, R., 2021. Dissemination of the *tet*(X)-variant genes from layer farms to manure-receiving soil and corresponding lettuce. Environ Sci Technol. 55(3), 1604-1614. https://doi.org/10.1021/acs.est.0c05042.
